# *Castanea sativa* (European Chestnut) Leaf Extracts Rich in Ursene and Oleanene Derivatives Block *Staphylococcus aureus* Virulence and Pathogenesis without Detectable Resistance

**DOI:** 10.1371/journal.pone.0136486

**Published:** 2015-08-21

**Authors:** Cassandra L. Quave, James T. Lyles, Jeffery S. Kavanaugh, Kate Nelson, Corey P. Parlet, Heidi A. Crosby, Kristopher P. Heilmann, Alexander R. Horswill

**Affiliations:** 1 Center for the Study of Human Health, Emory University, Atlanta, Georgia, United States of America; 2 Department of Dermatology, Emory University School of Medicine, Atlanta, Georgia, United States of America; 3 Department of Microbiology, Roy J. and Lucille A. Carver College of Medicine, University of Iowa, Iowa City, Iowa, United States of America; University Roma Tre, ITALY

## Abstract

The Mediterranean is home to a rich history of medical traditions that have developed under the influence of diverse cultures over millennia. Today, many such traditions are still alive in the folk medical practices of local people. Investigation of botanical folk medicines used in the treatment of skin and soft tissue infections led us to study *Castanea sativa* (European Chestnut) for its potential antibacterial activity. Here, we report the quorum sensing inhibitory activity of refined and chemically characterized European Chestnut leaf extracts, rich in oleanene and ursene derivatives (pentacyclic triterpenes), against all *Staphylococcus aureus* accessory gene regulator (*agr*) alleles. We present layers of evidence of *agr* blocking activity (IC_50_ 1.56–25 μg mL^-1^), as measured in toxin outputs, reporter assays hemolytic activity, cytotoxicity studies, and an *in vivo* abscess model. We demonstrate the extract’s lack of cytotoxicity to human keratinocytes and murine skin, as well as lack of growth inhibitory activity against *S*. *aureus* and a panel of skin commensals. Lastly, we demonstrate that serial passaging of the extract does not result in acquisition of resistance to the quorum quenching composition. In conclusion, through disruption of quorum sensing in the absence of growth inhibition, this study provides insight into the role that non-biocide inhibitors of virulence may play in future antibiotic therapies.

## Introduction

Alarming trends in the spread of antibiotic resistance among top pathogens, including *Staphylococcus aureus*, have placed mankind at the brink of what has been coined as the ‘post-antibiotic era’[1]. Since the widespread introduction of antibiotics in the 1940s, the same storyline has repeated itself over and over again: new antibiotic is introduced and then resistant variants emerge and quickly spread, effectively limiting the utility and lifespan of the drug. From an evolutionary biology perspective, this is not surprising; indeed, resistant mutants are expected to arise when any lifeform with the ability to rapidly reproduce and mutate is faced with a direct selective pressure, especially when a single drug is used against a single target. A new approach to antibiotic therapy is necessary. Many have proposed the strategy of an indirect attack on bacteria through interfering with their means of communication, also known as quorum sensing. Targeting microbial communication makes sense for a number of reasons, most importantly being that bacteria coordinate many of their virulence and pathogenesis pathways through these systems. Thus, ‘quorum quenchers’, or inhibitors of bacterial communication systems that are responsible for ‘collective decision making’[2] in microbes, could hold the key to pathogen disarmament, and improve therapeutic outcomes when used in conjunction with existing lines of antibiotics.

The majority of antibiotics used in modern medicine are natural products derived from soil microbes. Indeed, the soil has continued to be a center point of research in this field, and the source of some of the most recent antibiotic discoveries [3]. An underappreciated potential source of anti-infective natural products in modern medicine, however, is terrestrial plants. While mankind has a long and vibrant history of medical traditions involving plants in various traditional pharmacopoeia, our scientific understanding of the efficacy of plant based therapies and their respective mechanisms of action is still in its infancy. The limitations in identifying antibiotics from botanical sources may be linked to inherent problems in the very focus on bacteriostatic and bactericidal assays in the discovery process.

A series of studies by Quave et al. [4–6] investigated the bioactivity of plant extracts used in the traditional treatment of skin and soft tissue infections (SSTI) in Italy. Extracts were screened for activity against multiple targets, including *S*. *aureus* biofilms, communication (quorum-sensing) and growth. As a result of this work, three potential leads (*Castanea sativa*, *Ballota nigra*, and *Sambucus ebulus*) for the inhibition of quorum sensing in the absence of growth-inhibitory effects were identified [4]. Here, we continue to explore other mechanisms by which anti-infective traditional botanical medicines may function, and report the discovery of quorum quenching natural products extracted from *Castanea sativa* (European Chestnut) leaves, which are used in traditional therapies for treating skin inflammation SSTIs in the Mediterranean [7]. Notably, we report the ability of *C*. *sativa* leaf extracts to attenuate virulence by quenching *S*. *aureus agr-*mediated quorum sensing, effectively blocking production of harmful exotoxins at sub-inhibitory concentrations for growth. We also report the lack of cytotoxicity to human skin cells, lack of growth inhibitory activity against the normal skin microflora, lack of resistance development, and efficacy in a skin abscess animal model.

### Disarming an invasive, opportunistic pathogen

*Staphylococcus aureus* is an abundant, opportunistic pathogen that is the causative agent of numerous infections. Due to its prevalence as a leading cause of healthcare-associated infection, and its highly multidrug resistant nature, *S*. *aureus* is listed among pathogens included under the “serious threat” list by the CDC [1]. It colonizes the nasal passages of approximately 30% of the healthy adult population, which translates to 79 million colonized people in the US alone [8]. *S*. *aureus* infections initiate through trauma to the skin or mucosal layer and then progress through an invasive or toxin-mediated process. The prevalence of these infections has increased due to higher rates of colonization, immunosuppressive conditions, greater use of surgical implants, and dramatic increases in antibiotic resistance.

**Fig 1:**
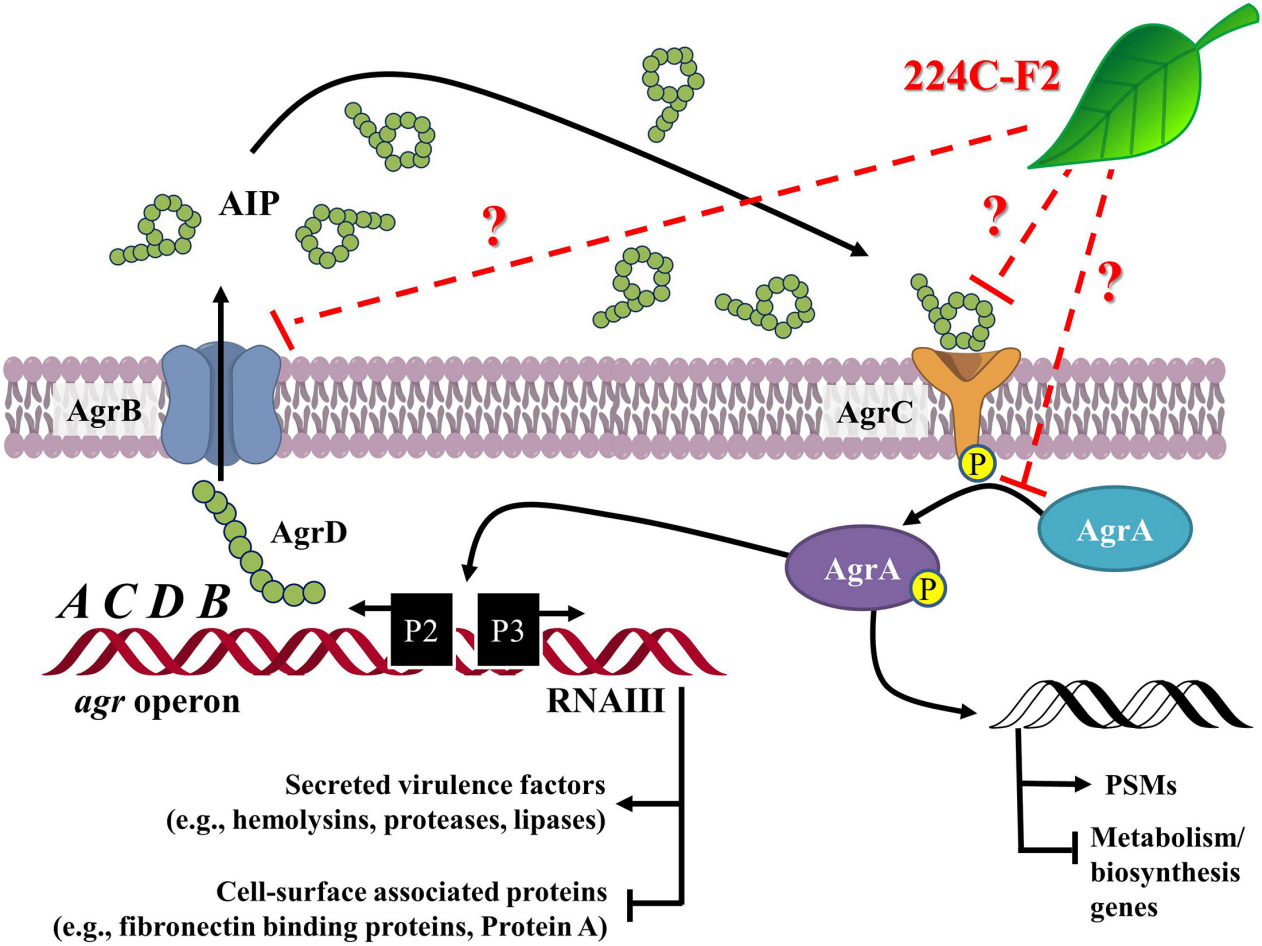
Schematic of the *Staphylococcus aureus* accessory gene regulator system. The *agr* locus has been investigated in detail and is known to contain two divergent transcripts named RNAII and RNAIII [9]. The RNAII transcript is an operon of four genes, *agrBDCA*, that encode factors required to synthesize AIP and activate the regulatory cascade. Briefly, AgrD is the precursor peptide of AIP, AgrB is a membrane protease involved in generating AIP, AgrC is a histidine kinase that is activated by binding AIP, and AgrA is a response regulator that induces transcription of both RNAII and RNAIII. The RNAIII transcript yields a regulatory RNA molecule that acts as the primary effector of the *agr* system by up-regulating extracellular virulence factors and down-regulating cell surface proteins [65]. The *agr* pathway is illustrated here with potential target sites for 224C-F2.

*S*. *aureus* produces an extensive array of enzymes, hemolysins, and toxins that are essential to its ability to spread through tissues and cause disease [9]. These virulence factors serve a wide scope of purposes in the infection process, including disruption of the epithelial barrier, inhibition of opsonization by antibody and complement, neutrophil cytolysis, interference with neutrophil chemotaxis, and inactivation of antimicrobial peptides [10–13]. The expression of all of these invasive factors is controlled by cell-density quorum sensing using the autoinducing peptide (AIP) molecule (Fig 1). Like other quorum-sensing signals, AIP accumulates outside the cell until it reaches a critical concentration and then binds to a surface receptor called AgrC, initiating a regulatory cascade. Since AIP controls the expression of accessory factors for *S*. *aureus*, this regulatory system has been named the accessory gene regulator (*agr*), and the majority of the proteins necessary for this quorum-sensing system to function are encoded in the *agr* chromosomal locus [9, 14]. Applying inhibitors to quench this communication system to attenuate pathogenicity and virulence lies at the core of this approach [15, 16].

*Agr* plays a key role in *S*. *aureus* pathogenesis. For example, SSTIs are the most common type of infection caused by *S*. *aureus* [17, 18]. These range from minor inflammatory conditions to more invasive infection, and most of these cases are associated with the formation of abscesses, the hallmark of a *S*. *aureus* infection. Through the use of genetic and *agr-*inhibiting tools, the *agr* system’s importance to abscess formation has been confirmed [19–23]. The bulk of the phenotype is due to *agr*-dependent secreted virulence factors as demonstrated with studies on sterile supernatants from wild type and *agr* mutant strains [20, 24, 25]. Interference with the *agr* system through the use of competing AIPs or AIP-sequestering antibodies decreased abscess formation [20, 21, 23]. These findings provide direct support for the notion that *agr*-targeted therapies could be an option for the development of skin infection treatments. Looking at other types of infections, *agr* mutants also display attenuated virulence in mice in the establishment of pneumonia and mortality [26–29], and in a systemic bloodstream infection model [30].

Given the importance of the *agr* system in pathogenesis, it has become the target of a number of anti-virulence chemical approaches [31]. With the extracellular exposure of the AgrC receptor, chemists have developed receptor antagonists that successfully inhibit the system *in vitro* and quench a *S*. *aureus* mouse skin infection [32–34]. Since there are different groups of the *agr* system (4 alleles), broad spectrum inhibitors were developed to extend the applicability of the antagonist. To the best of our knowledge, these leads were never pursued in a comprehensive way for therapeutic development, perhaps because they are labile synthetic peptides and possess poor bioavailability or pharmacokinetic properties. Other recent leads have included AgrA inhibitors, savirin [35] and the polyhydroxyanthraquinones [36, 37], AgrC antagonists solonamide A and B [38] and the AgrB inhibitor ambuic acid [39]. The present study represents the first in-depth analysis of botanical natural product inhibitors for *agr* first identified in the Quave et al. 2011 screening paper on quorum quenching Italian medicinal plants [4]. We hypothesize that by using a complex botanical composition to target quorum sensing rather than growth inhibition, the typical pitfalls of classical antibiotics can be avoided by limiting impact on the cutaneous microbiome and avoiding generation of resistance.

## Materials and Methods

### Collection and crude extraction of plant materials

Fresh leaves of the European Chestnut (*Castanea sativa* Mill., Fagaceae) were collected from wild populations in the months of May-July (2012–2014) in the Rionero-Alto Bradano region of the Basilicata Province in southern Italy following standard guidelines for collection of wild specimens [40]. Collections were made on private land with the permission of the landowner. Voucher specimens (CQ-309) were deposited at the *Herbarium Lucanum* (HLUC) at the *Universitá della Basilicata* in Potenza, Italy and the Emory University Herbarium (GEO) in Atlanta, GA, USA. The specimens were identified using the standard Italian Flora [41] and identification was confirmed at HLUC. European Chestnut leaves were shade-dried, ground with a blender, and vacuum sealed with silica packets prior to shipment to the US (under USDA permit P587-120409-008) for extraction and analysis. Upon arrival at the lab, leaves were further ground into a fine powder with a Thomas Wiley Mill at a 2 mm mesh size (Thomas Scientific).

### Extraction and purification of QSI-containing fractions

Crude methanol extracts (Extract 224) of the ground leaves were created by maceration of the plant materials at room temperature using a ratio of 1g dry leaves:10 mL MeOH for two successive periods of 72 hours, with daily agitation. Filtered extracts were combined, concentrated at reduced pressure and a temperature <40°C with rotary evaporators, and lyophilized before being re-suspended in water and partitioned in succession with hexane, ethyl acetate and butanol (all solvents acquired from Fisher Chemical, Certified ACS). The resulting non-aqueous partitions were dried over anhydrous Na_2_SO_4_, concentrated *in vacuo*, and lyophilized before testing for activity.

**Fig 2:**
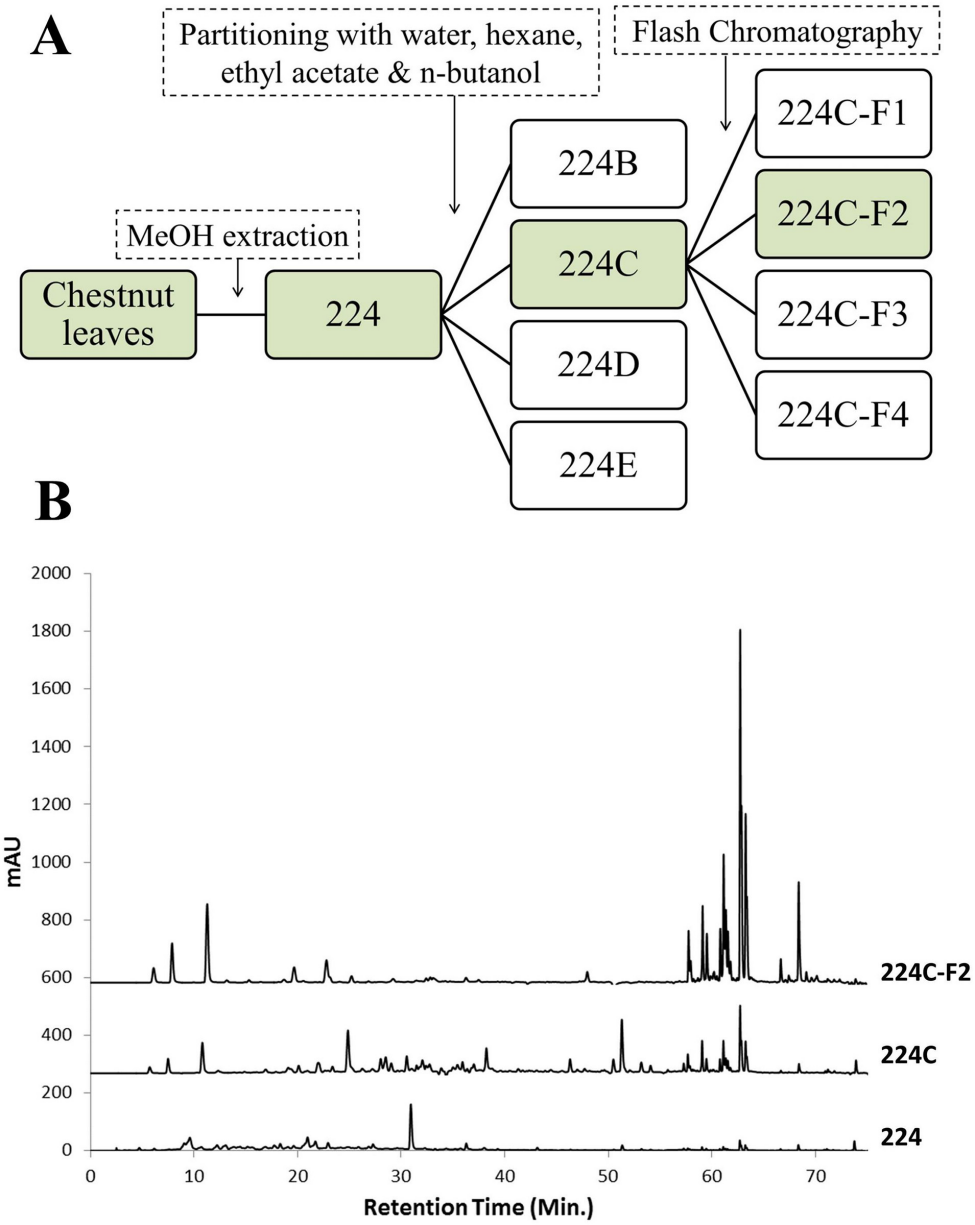
Isolation scheme. **(A)** The bioassay-guided fractionation scheme is illustrated, demonstrating the path from raw plant material to isolated, active natural products. (**B)** The corresponding HPLC chromatogram for the most active fractions illustrates how fractionation functions to increase the relative levels of active agents.

The most active partition (ethyl acetate, extract 224C) was subjected to further fractionation using a CombiFlash Rf+ (Teledyne ISCO) flash chromatography system using a RediSep Rf Gold silica column. Extract 224C was bonded to Celite 545 (Acros Organics) at a 1:4 ratio and dry-loaded using a RediSep dry load cartridge. The mobile phase consisted of (A) hexane, (B) EtOAc, and (C) MeOH. The linear gradient begins with 100% A for 6.3 column volumes (CV), and then increased to 50:50 A:B by25.3 CV, and increased to 100% B at 63.3 CV, which was held until 69.6 CV, and then to 70:30 B:C at 88.6 CV, which was held until 94.9 CV. The chromatography was monitored at 254 and 280 nm, as well as via ELSD. The resulting fractions were combined into 5 fractions. Following further bioassay testing, it was determined that the fraction which eluted from 30–40 CV (224C-F2) was most active. The full extract fractionation scheme is presented in Fig 2.

### Characterization by HPLC and LC-FTMS

An analytical HPLC-method was developed for the purposes of characterization of 224 and fractions. The analysis was performed on an Agilent 1260 Infinity system running OpenLab CDS ChemStation (Agilent Technologies, Santa Clara, CA, USA) with an Agilent ZORBAX Eclipse XDB-C18 (250 mm x 4.6 mm, 5 μm) column with compatible guard column at a column temperature of 40°C. Mobile phase reagents were HPLC-grade and purchased from Fisher Scientific, except for the Type 1 water, which was obtained from an EMD Millipore MILLI-Q water system (Billerica, MA). Mobile phase consisted of a linear gradient elution 0.1% formic acid in acetonitrile (A) and 0.1% formic acid in water (B) at a flow rate of 1 mL/min. Initial conditions were 98:2 (A:B) changing to 70:30 (A:B) at 50 min, to 2:98 (A:B) at 70 min and held until 85min., Samples were prepared in DMSO and 10 μL injections were made. Chromatograms were monitored at 254 nm and 314 nm.

Liquid chromatography-Fourier transform mass spectrometry (LC-FTMS) was performed on 224C-F2 using a Shimadzu SIL-ACHT and Dionex 3600SD HPLC pump with a modification of the previous chromatographic conditions. A 20 μL injection at ambient temperature with 0.1% formic acid in Optima LC/MS acetonitrile (Fisher Scientific) (A) and 0.1% formic acid in water (B) at a flow rate of 1 mL/min. Initial conditions were 98:2 (A:B) changing to 64:36 (A:B) at 12 min, to 52:48 (A:B) at 86 min, 2:98 (A:B) at 102.6 min and held until 117.6 min before returning to initial conditions to equilibrate the column. The data was acquired in MS^1^ mode scanning from a *m/z* of 150–1500 on a Thermo Scientific LTQ-FT Ultra MS in negative ESI mode and processed with Thermo Scientific Xcalibur 2.2 SP1.48 software (San Jose, CA). The capillary temperature was 275.0°C, sheath gas of 60, source voltage and current 5.0 kV and 100.0 μA, and the capillary voltage -49.0 V.

Putative compounds were determined for compounds present in the bioactive active region of 224C-F2’s chromatogram (retention time of 21–49 min). The Dictionary of Natural Products (CRC Press) and Scifinder (Chemical Abstracts Service) were searched in May 2015 using similar methodology. The high resolution mass of the compound was determined from the LC-FTMS data and the database searched for all compounds within ± 0.5 Da. The resulting compounds were limited to only those identified in the genus *Castanea*, for DNP several entries for the misspelling “Castaneae” was also included. The molecular formulas of the remaining compounds were compared to empirical formulas derived from the MS data and those that matched the experimental molecular mass with a delta of less than 100 ppm were evaluated further. Only small molecules were considered for further evaluation. Publications on the remaining small molecules were reviewed and the presence of the compound in the genus was verified.

In addition to examining LC-FTMS data and fragmentation patterns as described above, a number of natural products reported to occur in crude *C*. *sativa* leaf extracts [42] were specifically searched for in 224C-F2: chlorogenic acid, ellagic acid hyperoside, isoquercitrin and rutin. Standards of chlorogenic acid and ellagic acid (MP Biomedicals, Solon OH) and hyperoside (Chromadex, Irvine, CA) were run on the analytical HPLC method described above to determine retention times, the others were examined by MS fragmentation patterns and published UV-Vis spectra [43]. Standards were evaluated for purity via HPLC-DAD.

### Bacterial strains, plasmids, and culture media

*S*. *aureus* cultures were grown in Tryptic Soy Broth (TSB) or Tryptic Soy Agar (TSA). Cation-adjusted Mueller–Hinton broth (CAMHB) was used for minimum inhibitory concentration (MIC) testing of *S*. *aureus*. The bacterial strains and plasmids used in this study are described in Table 1. *Escherichia coli* cultures were grown in Luria-Bertani (LB) broth or on LB agar plates supplemented with 100 μg mL^-1^ ampicillin (Amp) as required for plasmid maintenance. *S*. *aureus* chromosomal markers or plasmids were selected for with 10 μg mL^-1^ of chloramphenicol (Cam) or erythromycin (Erm). *Staphylococcus warneri* cultures were grown in TSB or Brain-Heart Infusion (BHI) agar. *Micrococcus luteus* cultures were grown in nutrient broth or agar. *Streptococcus mitis*, *Streptococcus pyogenes*, *Corynebacterium amycolatum*, *Staphylococcus haemolyticus* and *Staphylococcus epidermidis* cultures were grown in BHI broth or TSA with 5% sheep blood. *Corynebacterium striatum* cultures were grown in TSB or TSA with 5% sheep blood. *Propionibacterium acnes* cultures were grown in Reinforced Clostridial Medium (RCM) broth or TSA with 5% sheep blood under static, anaerobic conditions generated by GasPak EZ Systems. Unless otherwise stated, all broth cultures were grown at 37°C with shaking at 250 rpm.

**Table 1:** Description of bacterial strains and plasmids used in this study.

Designation	Species	Other Characteristics*	Ref.
AH408; SA502A	*Staphylococcus aureus*	*agr* group II	[66]
AH430	*Staphylococcus aureus*	SA502a + pDB59 cmR, yfp reporter, *agr* group II	[47]
AH845	*Staphylococcus aureus*	agr group I	[48]
AH1263; LAC	*Staphylococcus aureus*	CA-MRSA, PFT USA300, *agr* group I	[48]
AH1677	*Staphylococcus aureus*	AH845 + pDB59 cmR, yfp reporter, *agr* group I	[47]
AH1747	*Staphylococcus aureus*	MW2 + pDB59 cmR, yfp reporter, *agr* group III	[47]
AH1872	*Staphylococcus aureus*	MN EV(407) + pDB59 cmR, yfp reporter, *agr* group IV	[47]
AH2759	*Staphylococcus aureus*	AH1263 *agr* P3:lux	[36]
AH3052	*Staphylococcus aureus*	AH1263 *Δspa*	[67]
F0392; HM-262	*Streptococcus mitis*	HMP, oral cavity isolate	
FS1; NR-13441	*Corynebacterium striatum*	Clinical isolate from Italy, 2005–2007	
MGAS15252; NR-33709	*Streptococcus pyogenes*	serotype M59, Group A *Streptococcus* (GAS)	
HL005PA2; HM-493	*Propionibacterium acnes*	HMP, skin isolate	
MN EV(407)	*Staphylococcus aureus*	*agr* group IV	[47]
MW-2	*Staphylococcus aureus*	*agr* group III	[68]
NIHLM001; HM-896	*Staphylococcus epidermidis*	HMP, 2008 skin isolate from alar crease from healthy volunteer	
NRS-116; NR-45922	*Staphylococcus haemolyticus*	Glycopeptide intermediate, 2002 surgical isolate	
NRS385; NR-46071	*Staphylococcus aureus*	HA-MRSA, PFT USA500, MLST ST8, SCC mecIV, *agr* group I, sea+, seb+	[69]
SK46; HM-109	*Corynebacterium amycolatum*	HMP, skin isolate on arm of healthy volunteer	
SK58; HM-114	*Micrococcus luteus*	HMP, skin isolate on arm of healthy volunteer	
SK66; HM-120	*Staphylococcus warneri*	HMP, skin isolate on arm of healthy volunteer	
UAMS-1	*Staphylococcus aureus*	MSSA, osteomyelitis isolate	[53]
UAMS-929	*Staphylococcus aureus*	isogenic *sarA* mutant of UAMS-1	

*Other Characteristics: agr: accessory gene regulator; CA-MRSA: community-associated methicillin resistant *Staphylococcus aureus*; HA-MRSA: healthcare-associated MRSA; HMP: Human Microbiome Project isolate; PFT: pulsed field type; MLST: multilocus sequence type; MSSA: methicillin sensitive *Staphylococcus* aureus; SCC: staphylococcal chromosomal cassette; sea: staphylococcal enterotoxin A; seb: staphylococcal enterotoxin B; ST: sequence type; yfp: yellow fluorescent protein.

### Minimum inhibitory concentration (MIC)

Extract 224 and fractions were examined for minimum inhibitory concentrations (MIC) against strains representing the four *agr* alleles (AH430, AH1677, AH1747, AH1872), biofilm test strain (UAMS-1) and a USA500 strain (NRS385), which was used in δ-toxin quantification experiments. Clinical Laboratory Standards Institute (CLSI) M100-S23 guidelines for microtiter broth dilution testing were followed [44]. Controls include the vehicle, and antibiotics: Kanamycin (Kan) and Amp (MP Biomedicals Inc). All concentrations were tested in triplicate and repeated twice on different days. Briefly, overnight cultures in CAMHB were standardized by OD to 5 x 10^5^ CFU/mL, and this was confirmed by plate counts. Two-fold serial dilutions were performed on a 96-well plate (Falcon 35–1172) to achieve a test range of 512–0.25 μg mL^-1^ for extracts and 64–0.03125 μg mL^-1^ for Amp and Kan. Plates were incubated at 37°C for 18 hrs. under static conditions. Plates were read at an OD 600nm in a Cytation 3 multimode plate reader (Biotek) at 0 and 18 hrs. post inoculation. The following formula, which takes into account the impact of extract color and vehicle on the OD, was used as previously described [5]:

$$\%\;Inhibition=\left[1-\left(\frac{OD_{t18}-OD_{t0}}{OD_{vc18}-OD_{vc0}}\right)\right]\;\times 100$$

with OD_t18_ = OD of the test well at 18 hrs., OD_t0_ = OD of the test well at 0 hrs., OD_vc18_ = OD of the vehicle control well at 18 hrs, and OD_vc0_ = OD of the vehicle control well at 0 hrs. MIC_50_ and MIC_90_ values were assigned based on the concentration at which at least 50 or 90% inhibition of growth was observed as determined by OD, respectively.

Growth inhibition of the refined extract, 224C-F2, was also assessed for impact on the normal skin microflora. In all cases, with the exception of *P*. *acnes*, the appropriate CLSI method for MIC determination by broth microdilution was employed. Briefly, MICs for *Staphylococcus warneri*, *S*. *epidermidis*, *S*. *haemolyticus* and *Micrococcus luteus* were determined using the above described M100-S23 CLSI method [44] for *S*. *aureus* with vehicle and antibiotic controls. Amp and Kan (MP Biomedicals Inc) were used in all staphylococcal tests; Amp, Erm (Sigma Aldrich) and clindamycin, Clin (MP Biomedicals) were used for *M*. *luteus* controls. MICs for *Streptococcus pyogenes* and *S*. *mitis* were determined using the M100-S23 CLSI method [44] in CAMHB with 3% lysed horse blood (LHB), incubated at 37°C for 24 hrs under static conditions, with Amp and Erm as antibiotic controls. MICs for *Corynebacterium striatum* and *C*. *amycolatum* followed the M45-A2 CLSI method [45] in CAMHB with 3% LHB, incubated at 35°C for 24 hrs under static conditions, with Amp and Erm as antibiotic controls. MICs for *Propionibacterium acnes* were based on a previous method [46] using BHI supplemented with 1% dextrose, incubated at 37°C for 72 hrs under static, anaerobic conditions.

### Quorum quenching assays with reporter strains

Extracts were tested for quorum quenching activity against all four *agr* types using previously described [47] *agr* P3*-*YFP reporter strains AH1677 (type I), AH430 (type II), AH1747 (type III), and AH1872 (type IV), as well as previously described *agr* P3*-lux* (type I) reporter strain AH2759 [36]. Overnight cultures of reporter strains that were grown in TSB supplemented with Cam were inoculated at a dilution of 1:250 into fresh TSB containing Cam. 100 μL aliquots were added to 96-well microtiter plates (Costar 3603) containing 100 μL aliquots of TSB containing Cam and 2-fold serial dilutions (0.1–200 μg mL^-1^) of extracts 224, 224C, and 224C-F2. After mixing, the effective inoculum dilution was 1:500 and the final extract concentrations ranged from 0.05–100 μg mL^-1^, with a final DMSO concentration of 1% (v/v) in all wells. Four dilution series were prepared for each reporter/extract combination, and in addition 4 mock vehicle (DMSO) dilution series were included for each reporter strain. Microtiter plates were incubated at 37°C with shaking (1000 rpm) in a Stuart SI505 incubator (Bibby Scientific, Burlington, NJ) with a humidified chamber. Fluorescence (top reading, 493 nm excitation, 535 nm emission, gain 60) and optical density (OD) readings at 600 nm, or luminescence and OD_600_ readings in the case of reporter AH2759, were recorded at 30 min increments using a Tecan Systems (San Jose, CA) Infinite M200 plate reader.

### Hemolytic activity by red blood cell lysis assay

The quorum quenching activity of extracts was assessed by measuring the hemolytic activity of culture supernatants on rabbit red blood cell lysis. Overnight cultures of an Erm sensitive variant of USA300 strain LAC, AH1263 [48] and an *hla*::*Tn551* (AH1589) mutant of AH1263 [49] were inoculated 1:500 into 5 ml of TSB (in 17x150 mm culture tubes) containing extracts 224, 224C, or 224CF2 at concentrations of 6.25, 12.5, 25, 50 and 100 μg mL^-1^. In all tubes containing extract the mock vehicle (DMSO) concentration was held constant at 1% (v/v). Vehicle control tubes containing 1% DMSO were similarly prepared for AH1263, AH1589 well as for an Δ*agr*::*tetM* (AH1292) mutant of AH1263 [49]. All tubes were incubated at 37°C with shaking (250 rpm), and growth was monitored by periodically transferring 100 μL of culture to a 96-well microtiter plate and reading OD_600_ in a Tecan Systems (San Jose, CA) Infinite M200 plate reader. Following 6 hrs of incubation, 600 μL of each culture was filter sterilized using cellulose acetate SpinX 0.22 μm filters (Corning).

To quantify hemolytic activity, the filter sterilized culture supernatants were serially diluted in 2-fold steps (from 0.04–100%) in TSB, and 50 μL aliquots were dispensed in quadruplicate into 96-well microtiter plates. Rabbit erythrocytes, prepared from defibrinated blood (Hemostat Laboratories, Dixon, CA) by washing 3 times with 1.1x PBS and resuspending in 1.1x PBS at 1% (v/v), were added to the microtiter plates at 50 μL per well (yielding a final erythrocyte concentration of 0.5% (v/v)). The erythrocytes and culture supernatants were mixed thoroughly and incubated statically at room temperature for 2 hrs. Hemolysis was detected by the loss of turbidity as measured at OD_630_ using a Tecan Systems (San Jose, CA) Infinite M200 plate reader. Relative hemolytic activities were obtained by using KaleidaGraph 4.1.3 (Synergy Software, Reading, Pa., USA) to perform 4-parameter logistic fits of the turbidity data in order to determine the concentration of supernatant that resulted in 50% red blood cell lysis.

### Western blot for alpha-hemolysin

An overnight culture of *S*. *aureus* AH3052 *Δspa* was inoculated into 5 mL of TSB at 1:500 and grown at 37°C with shaking (250 rpm), in the presence of either DMSO or one of the extracts (224, 224C or 224C-F2) at concentrations of 6.25, 12.5, 25, 50 and 100 μg mL^-1^. Following 8 hours of incubation, 600 μL of each culture was filter sterilized using a cellulose acetate SpinX 0.22 μm filter (Corning) and the filter sterilized media was stored at -20^°^C. The filtered media was electrophoresed on 13% SDS-PAGE gels and transferred to nitrocellulose membranes (Bio-Rad). Membranes were blocked overnight at 4°C in TBST (20 mM Tris [pH 7.5], 150 mM NaCl, 0.1% Tween 20) with 5% nonfat dry milk then washed 3 times with TBST. Hla was detected using a polyclonal rabbit anti-Hla antibody (Shlievert Lab, University of Iowa) at a 1:5000 dilution and a goat anti-rabbit HRP secondary antibody (Jackson ImmunoResearch Laboratories) at a 1:20000 dilution. Blots were incubated at RT for 5 min with Supersignal West Pico Chemiluminescent Substrate (Thermo Scientific) then exposed to film for 30 min.

### Quantification of δ-toxin by HPLC

Overnight cultures of *S*. *aureus* NRS385 were standardized by OD to a starting density of 5 x 10^5^ CFU mL^-1^ in TSB, and this was verified by plate counts. The standardized culture was added to 14 mL test tubes containing the extract or vehicle control, for a final tube to volume ratio of 1:10. All extracts were examined at sub-MIC_50_ concentrations to avoid impact of growth inhibition on quorum sensing. Cultures were incubated at a 45° angle at 37°C while shaking (275 rpm) for 15 hrs, and then placed on ice until cultures were centrifuged (13,000 rcf x 5 min) into a pellet using a bench-top refrigerated (4°C) centrifuge. Supernatants were carefully removed and sterile filtered with a 0.22 μm nylon syringe filter (Membrane Solutions, Dallas, TX). Each supernatant was divided into equal aliquots for freezing at -20°C until needed for HPLC quantification of δ-toxin, toxicity testing on HaCaT cells and AIP I quantification.

Frozen supernatant samples were defrosted to room temperature and transferred to HPLC autosampler vials. Resolution of the de-formylated and formylated δ-toxin peaks was achieved on an Agilent 1260 Infinity system with a Resource PHE 1-mL (GE Healthcare, Uppsala, Sweden) analytical column, as previously described [4, 50]. Briefly, 500 μL of supernatant was injected onto the column. The toxins were eluted at a flow rate 2 mL min^-1^ using a gradient of two solvent systems: (A) 0.1% trifluoracetic acid (TFA) in water and (B) 0.1% TFA in acetonitrile (ACN). The mobile phase was 10% B for 3 min., 90% B for 7.5 min., 100% B for 2 min. and 0% B for 2 min. Peak integration was at 214 nm, with de-formylated and formylated δ-toxin recorded at a retention time of 6.4 and 6.8 min, respectively. Total peak height and areas were recorded. Peak identities were confirmed by running the same chromatographic method on the previously described LC-FTMS system in negative ESI mode and comparing the de-formylated and formylated δ-toxin ions to published values [51].

### Resistance passaging

To determine the ability of *S*. *aureus* to generate resistance to the quorum quenching effects of 224C-F2, cultures were exposed to sub-MIC concentrations (16 μg mL^-1^) of extract for 15 hrs, as described above, the OD_600_ taken, and cultures centrifuged. The cell-free supernatant was removed and frozen for later HPLC quantification of δ-toxin as described above. The cell pellets were then reconstituted in TSB to an OD equivalent of 5 x 10^5^ CFU mL^-1^ with extract (or vehicle control) added, and incubated while shaking as described above. This process was repeated for a total of 15 passaging days.

### Biofilm assay

Extract 224 and fractions were examined for impact on *S*. *aureus* biofilm formation using a human plasma protein-coated assay as previously described [6, 52] using strains UAMS-1 [53] (a PFGE USA200 osteomyelitis isolate, *agr* type III) and its isogenic *sarA* mutant, UAMS-929, which has a biofilm deficient phenotype and serves as a positive control. We also included the natural product-based anti-biofilm composition “220D-F2”, which has been shown to inhibit biofilm formation in both *Staphylococcus aureus* [6] and *Streptococcus pneumoniae* [54], as a positive drug control. Briefly, following inoculation and addition of appropriate media (containing extract or vehicle alone), 96-well plates (Falcon 35–1172) were incubated for 22 hrs at 37°C. The wells were gently washed with phosphate-buffered saline (PBS), fixed with ethanol, stained with crystal violet, rinsed in tap water, and the stain eluted into ethanol and transferred to a new plate prior to quantification of the eluate at an OD_595_ with a Cytation 3 multimode plate reader (Biotek).

### Human keratinocyte toxicity

Human immortalized keratinocytes (HaCaT cell line) were maintained in Dulbecco’s modified Eagle’s medium with L-glutamine and 4.5 g L^-1^ glucose (Corning, Corning, NY) supplemented with 10% heat-inactivated fetal bovine serum (Seradigm, Randor, PA) and 1X solution of 100 IU Penicillin and 100 μg mL^-1^ Streptomycin (Corning, Corning, NY) at 37°C, 5% CO_2_ in 75 cm^2^ flasks (Greiner Bio-One). Upon reaching suitable confluency (90–95%), cells were detached from the flask bottom for cell splitting and plating using 0.25% typsin, 0.1% ethylenediaminetetraacetic acid (EDTA) in Hanks' balanced salt solution (HBSS) without Ca++, Mg++ and NaHCO_3_ (Corning, Corning, NY). Toxicity of extracts and filtered spent bacterial supernatant from *S*. *aureus* (NRS385) (described in δ-toxin method above) were evaluated with the LDH Cytotoxicity assay (G-Biosciences, St. Louis, MO). Briefly, the cell culture was standardized to 4 x 10^4^cells mL^-1^ using a hemocytometer and 200 μL added per well in a 96 well tissue culture treated microtiter plate (Falcon 35–3075). Plates were incubated for 48 hrs to allow for seeding, prior to media aspiration. Either media containing extracts or vehicle were serially diluted 2-fold (0.25–512 μg mL^-1^) or media containing 20% (v/v) spent bacterial supernatant was added and were processed 24 hrs later following manufacturer’s protocol for chemical induced cytotoxicity.

The cytotoxic effects of bacterial supernatants and a positive control, Staurosporine (Sigma), were further examined with the Viability/Cytotoxicity Assay Kit (Biotum, Hayward, CA). Cells were plated in 24-well plates with glass coverslips. Cells were plated and grown to 90–95% confluence glass coverslips in 24 well plates (Costar 3526) before the addition of treatments. Cells were either treated with 14% (v/v) spent bacterial supernatants or 7.1μM staurosporine for 3 hrs, and then stained following manufacturer’s fluorescence microscopy protocol. After staining, the glass slides were mounted using ProLong Gold and fluorescence was assessed using a DMRXA2 microscope (Leica) with narrow band pass Texas Red and FITC filters. Images were collected with ORCA-ER digital camera (Hamamatsu) and processed using Simple PCI software (Hamamatsu) and ImageJ software (National Institutes of Health Research Services Branch, Bethesda, MD, USA).

### Mice and *S*. *aureus* skin infection model

C5Bl/6 dams were purchased from Charles Rivers (Wilmington, MA). Mice were allowed to acclimate to the BSL-2 level animal housing facility at the University of Iowa (Iowa City, IA) for at least seven days, prior to their inclusion in this study. All animal work described herein was approved by and conducted in accordance with the recommendations of Animal Care and Use Committee at the University of Iowa (IACUC # 1205097). At D0, 8–12 week old mice were anesthetized with isoflurane, abdominal skin was carefully shaved with an Accu-Edge microtome blade (Sakura-Finnetek, Torrance, CA) and exposed skin was cleansed by wiping with an alcohol prep pad (Covidien, Mansfield, MA). For inoculum preparation, a USA 300 MRSA strain (AH1263) or its deletion mutant (AH1292) were grown in TSB medium overnight at 37°C in a shaking incubator set to 200 rpm. Log-phase bacteria were obtained after a 2 hr subculture of a 1:100 dilution of the overnight culture in TSB. Bacterial cells were pelleted and resuspended in DPBS to a concentration of 1x10^8^ CFUs/45 μL. 50 μL inoculum suspensions containing 1x10^8^ CFUs and either 224C-F2 (5 μg, or 50 μg diluted in DMSO) or DMSO alone were injected to intradermally into abdominal skin using 0.3 mL/31 gauge insulin syringe (BD, Franklin Lakes, NJ). Infectious dose was confirmed by plating serial dilutions of inoculum on TSA and counting ensuing colonies after overnight culture. Baseline body weights of mice were measured before infection and every day thereafter for a period of 7 days. For determination of lesion size, digital photos of skin lesions were taken daily with a Canon Rebel Powershot (ELPH 330 HS) and analyzed via ImageJ software (National Institutes of Health Research Services Branch, Bethesda, MD, USA). Following infection, mice were monitored daily for signs of overt distress that had been pre-established as humane endpoint criteria *e*.*g*., weight loss exceeding 20% of baseline (D0) body weight, hunching, loss of mobility and ruffled fur. As no such signs of distress were observed in the present study, all animals were euthanized *via* continuous administration of 100% CO_2_ at the experimental end point.

### Statistical analysis

All tests were performed in triplicate and repeated on at least two different occasions. Pair-wise testing was performed based on the Student’s *t* test in Microsoft Excel.

## Results

### Isolation of a highly bioactive fraction: 224C-F2

Fractionation of the crude *Castanea sativa* leaf extract (224) was guided by measures of bioactivity, selecting for fractions that exhibited quorum quenching with little to no growth inhibitory activity, Fig 2. This was measured through use of reporter strains for *agr* types I-IV. To create fractions for testing, extract 224 was suspended in water and partitioned in succession using hexane, ethyl acetate and butanol. The ethyl acetate partition (224C) was determined to be the most bioactive under these testing parameters and was selected for further fractionation with a flash chromatography system using a gradient of hexane, ethyl acetate and methanol. The most active fraction (224C-F2) was selected for further testing and chemical characterization, described below.

### 224C-F2 inhibits *S*. *aureus* quorum sensing across the diversity of *agr* alleles

**Fig 3:**
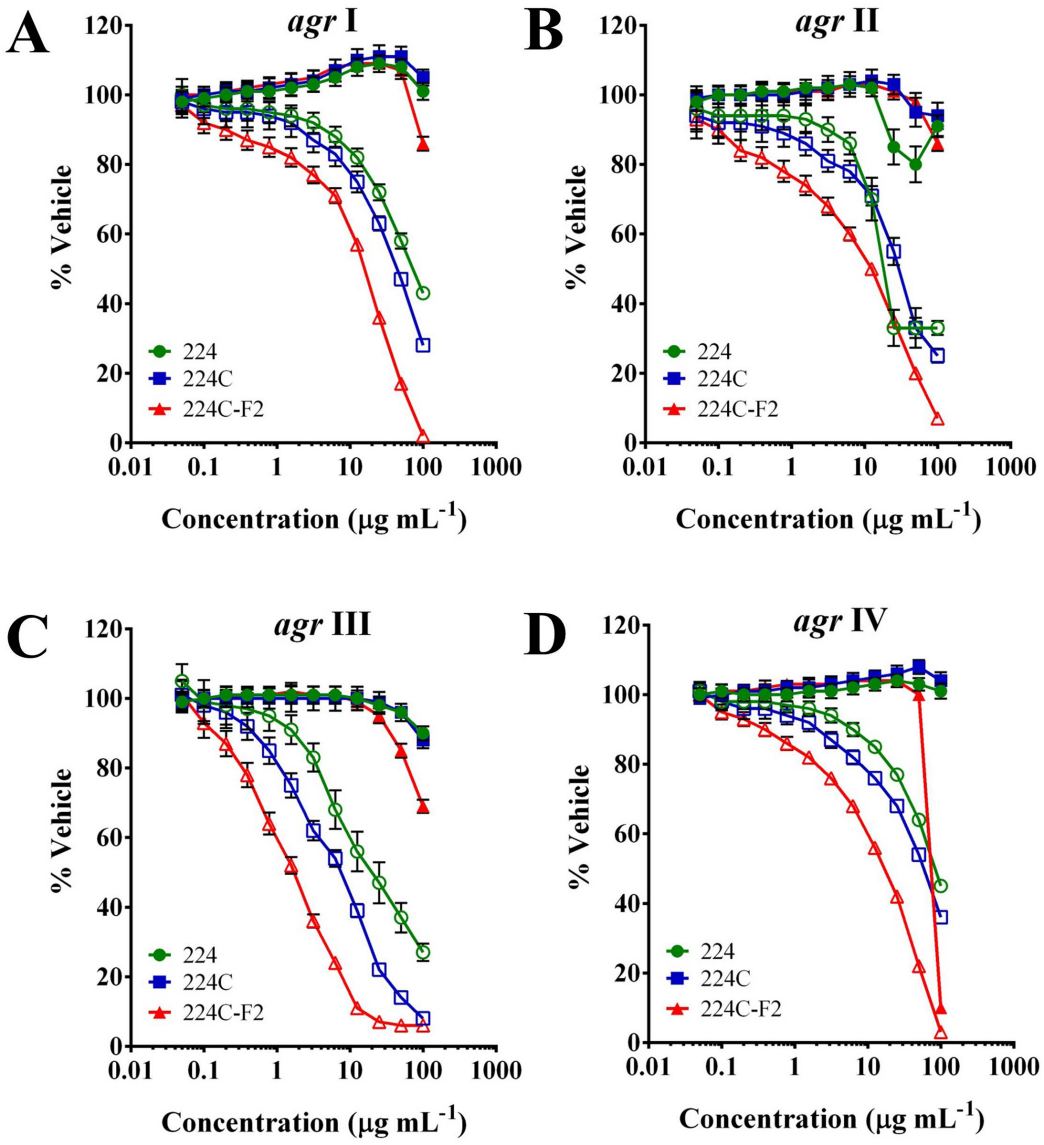
European Chestnut leaf extracts inhibit all four *S*. *aureus agr* alleles a non-biocide manner. *S*. *aureus agr* reporter strains were treated with extracts 224, 224C, and 224C-F2 at a dose range of 0.05–100 μg mL^-1^. Bioactivity guided sequential fractionation resulted in increased quenching of all 4 *agr* alleles in a manner independent of growth inhibition. Optical density of the culture is represented by solid black symbols; fluorescence in the *agr* reporters is indicated by the open symbols. The IC_50_ and IC_90_ for quorum quenching impact of each extract are reported in Table 3. **(A)** agr I, AH1677; **(B)** agr II, AH430; **(C)** agr III, AH1747; **(D)** agr IV, AH1872.

A number of *in vitro* assays were employed to guide fractionation of the natural product composition and to evaluate efficacy in blocking *S*. *aureus* quorum sensing mediated virulence. Growth inhibitory impact of the extracts was assessed with traditional static MIC assays (Table 2); growth inhibition was also tracked in the fluorescent reporter assays for *agr* activity (Fig 3). A slightly higher level of growth inhibition was observed in the static MIC assays over that observed in the super-aerated reporter assay, but in all reporter strains, the MIC remained >100 μg mL^-1^ for 224C-F2. Limited biofilm inhibitory activity of the extracts was noted (Table 2).

**Table 2:** Growth and biofilm inhibition studies. Minimum inhibitory concentrations (MIC) were determined for extracts 224, 224C, 224C-F2 and control antibiotics (Ampicillin and Kanamycin) against *Staphylococcus aureus* strains. Minimum biofilm inhibiting concentration (MBIC) determination is also presented, and compared to control extract 220D-F2. All MIC and MBIC values are represented in μg mL^-1^.

Strain ID	MIC	Test Agent (μg mL^-1^)					
		224	224C	224C-F2	Amp	Kan	220D-F2
**AH430**	MIC_50_	64	64	64	0.0625	2	—
	MIC_90_	ND	ND	ND	0.125	4	—
**AH1677**	MIC_50_	32	16	64	ND	ND	—
	MIC_90_	ND	ND	256	ND	ND	—
**AH1747**	MIC_50_	128	16	8	ND	1	—
	MIC_90_	ND	ND	256	ND	2	—
**AH1872**	MIC_50_	16	64	16	4	1	—
	MIC_90_	ND	ND	128	8	4	—
**NRS385**	MIC_50_	16	16	16	ND	ND	—
	MIC_90_	ND	ND	128	ND	ND	—
**UAMS-1**	MIC_50_	32	64	32	ND	2	128
	MIC_90_	ND	ND	ND	ND	4	ND
	MBIC_50_	200	100	200	—	—	12.5
	MBIC_90_	ND	ND	400	—	—	100

**ND:** MIC not detected at the concentration range tested (0.25–512 μg mL^-1^ for extracts; 64–0.03125 μg mL^-1^ for ampicillin and kanamycin).

**—:** Not tested.

Quorum quenching effects for 224C-F2 were observed at IC_50_ values of 1.56–25 μg mL^-1^, depending upon the strain tested (Table 3). The most potent quorum quenching activity was observed for *agr* III (IC_50_ of 1.56 μg mL^-1^), and the least for *agr* IV (IC_50_ of 25 μg mL^-1^). Significant inhibition of *agr* was observed for all *agr* alleles at sub-inhibitory concentrations for growth, indicating that the quorum-quenching activity is due to specific interference with *agr*, and not simply the result of a false positive due to growth inhibition.

**Table 3:** Inhibition of *S*. *aureus* quorum sensing *by Castanea* sativa leaf extracts as detected by *agr* reporter strains. All tests were performed at sub-MIC_50_ concentrations to avoid data skewing from potential growth inhibition effects. All IC values are represented in μg mL^-1^.

Strain ID	*agr* group	IC	Test Agent (μg mL^-1^)		
			224	224C	224C-F2
AH1677	I	IC_50_	100	50	25
		IC_90_	ND	ND	100
AH430	II	IC_50_	25	50	12.5
		IC_90_	ND	ND	100
AH1747	III	IC_50_	25	12.5	1.56
		IC_90_	ND	100	12.5
AH1872	IV	IC_50_	100	100	25
		IC_90_	ND	ND	100

**ND:** IC not detected at the concentration range tested (0.05–100 μg mL^-1^).

**Fig 4:**
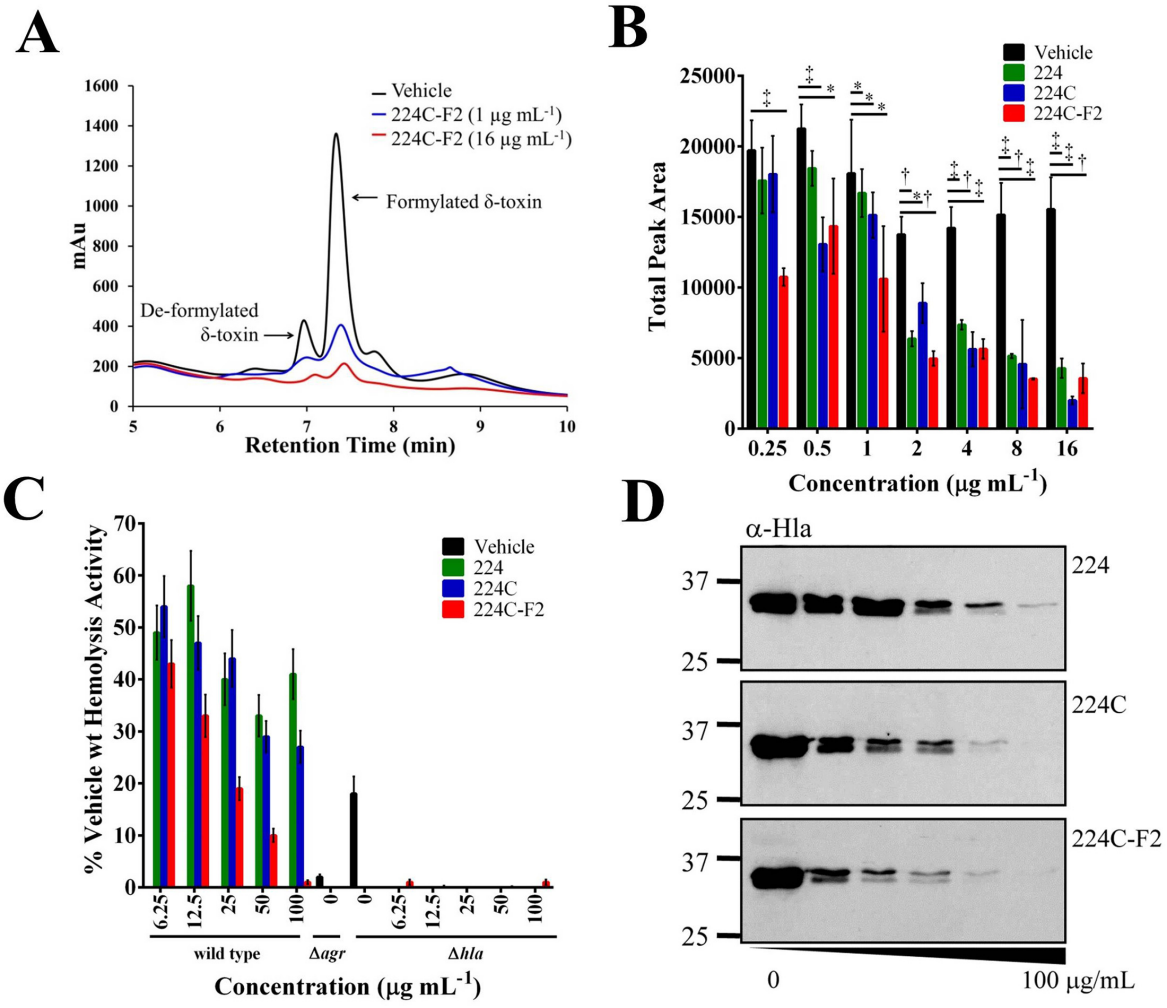
224C-F2 blocks MRSA exotoxin production. **(A)** 224C-F2 demonstrates a dose-dependent effect in inhibition of de-formylated and formylated delta toxin, as illustrated in this HPLC chromatogram. **(B)** Quantification of delta-toxin confirmed the dose-dependent inhibitory activity of extracts, and the increased activity of the refined fraction 224C-F2 over 224 and 224C. **(C)** Extracts quench the hemolytic activity of both the *S*. *aureus* wild type and Δ*hla* mutant, demonstrating that in addition to preventing production of α-hemolysin (responsible for the major share of hemolytic activity), that extracts also inhibit PSM production, responsible for the observable hemolytic activity in *hla* mutant strains. All treated groups are significant in comparison to the vehicle control (*p*<0.001). **(D)** USA300 (Δ*spa*) was exposed to increasing doses of 224, 224C, 224C-F2, and vehicle control for 8 hrs. Western blot for α-hemolysin on supernatants demonstrated a dose-dependent decline in protein levels. Significant differences between treatment and vehicle are represented as: *: *p*<0.05; ‡: *p*<0.01; †: *p*<0.001.

To verify the observed quorum quenching activity, downstream translational products of the quorum sensing system were assessed. HPLC quantification of δ-toxin (Fig 4A) from the supernatant of a heavy producer of exotoxins (NRS385, a USA 500, *agr* I, HA-MRSA isolate) revealed significant reduction (p<0.01) in production of δ-toxin in 224C-F2 treated cultures at doses as low as 0.25 μg mL^-1^ (Fig 4B).

To verify the block in production of additional exotoxins, cultures of strain LAC (AH1263, a USA300, *agr* I, CA-MRSA isolate) and its isogenic *agr* (AH1292) and *hla* (AH1589) mutants were grown in the presence of the extracts and their supernatants were examined in a rabbit red blood cell lysis assay. In this assay, the majority of RBC lysis is attributed to the presence of α-hemolysin in the culture supernatant. The presence of some lytic activity in the Δ*hla* vehicle control suggests that some additional hemolytic activity (~18%) may be due to additional toxins in the supernatant, phenol soluble modulins (PSMs), in particular. Treatment of wild type with 224C-F2 resulted in significant (*p*<0.001) reduction in hemolytic activity in wild type strain at 6.25 μg mL^-1^, and almost total loss of hemolytic activity at the concentration of 100 μg mL^-1^. Treatment of the Δ*hla* mutant demonstrated nearly total loss of hemolytic activity at 6.25 μg mL^-1^ (Fig 4C). Similar to the hemolysis assessment, when USA300 is exposed to increasing doses of all extracts (224, 224C, and 224C-F2), the level of α-hemolysin protein production is markedly attenuated, with the most potent activity exhibited by 224C-F2 (Fig 4D).

### 224C-F2 blocks *S*. *aureus* damage to human keratinocytes

**Fig 5:**
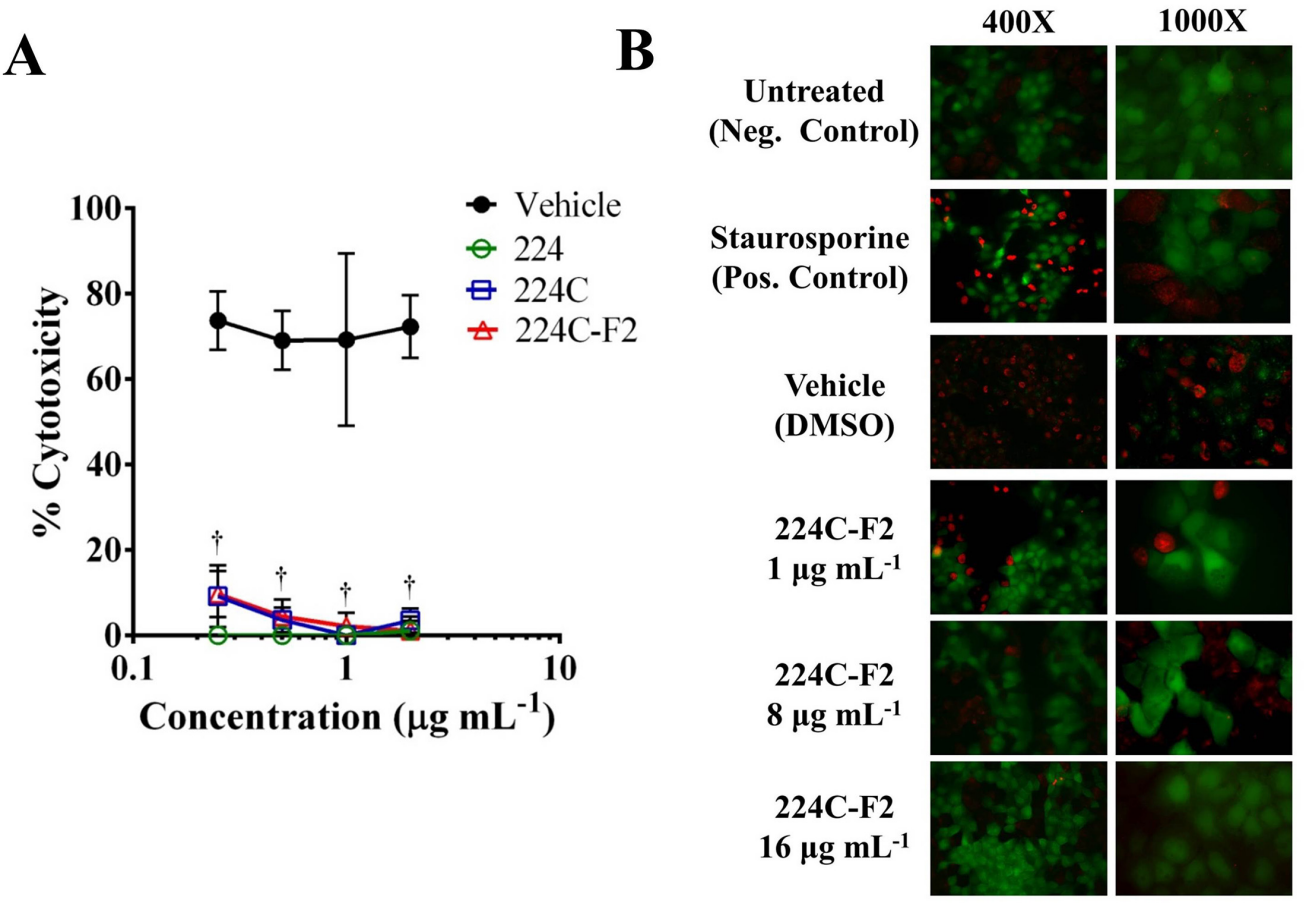
Spent supernatants of *S*. *aureus* treated with 224C-F2 exhibit diminished cytotoxic effects against human keratinocytes. **(A)** Supernatants were applied to HaCaT cells (20% v/v for 24 hrs) to measure the lytic capacity (determined by LDH assay) of a full suite of *S*. *aureus* exotoxins. Supernatants from 224C-F2-treated cultures were non-toxic to the mammalian cells, confirming inhibition of exotoxin production. **(B)** Following exposure to supernatants (14% v/v for 3 hrs) or staurosporine (7.1 μM for 3 hrs), HaCaT cells were imaged by fluorescent microscopy to examine cell integrity. Green cells are live, red are dead. Black regions are indicative of dead cells that have detached from the slide. Significant differences between treatment and vehicle are represented as: *: *p*<0.05; ‡: *p*<0.01; †: *p*<0.001.

In addition to monitoring the activity of each *agr* allele and detecting specific downstream products (e.g. α-hemolysin and δ-toxin), we also broadened our scope to capture virulence impact data on any other exotoxins that could be produced through this system. To do this, we exposed HaCaT cells to the sterile-filtered supernatants of treated and control cultures. The difference in cytotoxicity as detected by LDH assay was very clear (*p*<0.001) for all extracts (224, 224C, and 224C-F2) in comparison to control, and this was evident at doses as low as 0.25 μg mL^-1^ (Fig 5A). Likewise, images of the HaCaT cells following exposure to the supernatants reaffirmed the lack of exotoxins in the supernatants in 224C-F2 treated cultures (Fig 5B).

### 224C-F2 does not inhibit the growth of common skin bacteria

We investigated the potential of 224C-F2 to create a state of dysbiosis by inhibiting the growth of specific members of the normal skin microflora. While our studies were restricted to assessing the MICs of Actinobacteria and Firmicutes, we did find that 224C-F2 has little to no growth inhibitory activity against the Actinobacteria (*Corynebacterium amycolatum*, *C*. *striatum*, *Micrococcus luteus*, and *Propionibacterium acnes*) and Firmicutes (*Staphylococcus epidermidis*, *S*. *haemolyticus*, *S*. *warneri*, *Streptococcus mitis*, and *S*. *pyogenes*) tested (Table 4) at the concentrations required for quorum quenching activity in *S*. *aureus*. Of these species, *S*. *warneri* was the most sensitive, with an MIC_50_ of 32 μg mL^-1^; the MIC_90_ was not detectable at the range tested (4–512 μg mL^-1^).

**Table 4:** 224C-F2 has limited impact on growth of common skin microflora. Minimum inhibitory concentration (MIC) determination for 224C-F2 and antibiotic controls (ampicillin, erythromycin, clindamycin and kanamycin) against bacterial skin microflora. All MIC values are represented in μg mL^-1^.

			Test Agent (μg mL^-1^)				
Species	Strain ID	MIC	224C-F2	Amp	Erm	Clin	Kan
*Corynebacterium amycolatum*	SK46	MIC_50_	ND	0.125	0.125	-	-
		MIC_90_	ND	ND(64)	ND(64)	-	-
*Corynebacterium striatum*	FS1	MIC_50_	ND	ND(16)	ND(8)	-	-
		MIC_90_	ND	ND(16)	ND(8)	-	-
*Micrococcus luteus*	SK58	MIC_50_	64	0.25	0.125	0.25	-
		MIC_90_	128	0.5	0.125	0.5	-
*Propionibacterium acnes*	HL005PA2	MIC_50_	128	-	0.0625	0.0625	-
		MIC_90_	ND	-	0.125	0.25	-
*Staphylococcus epidermidis*	NIHLM001	MIC_50_	64	0.03125	-	-	1
		MIC_90_	128	0.0625	-	-	1
*Staphylococcus haemolyticus*	NRS116	MIC_50_	ND	ND(16)	ND(32)	-	-
		MIC_90_	ND	ND(16)	ND(32)	-	-
*Staphylococcus warneri*	SK66	MIC_50_	32	0.03125	-	-	0.25
		MIC_90_	ND	0.125	-	-	1
*Streptococcus mitis*	F0392	MIC_50_	ND	0.03125	0.03125	-	-
		MIC_90_	ND	0.0625	0.03125	-	-
*Streptococcus pyogenes*	MGAS15252	MIC_50_	ND	0.03125	0.03125	0.0625	-
		MIC_90_	ND	0.125	0.0625	0.125	-

**ND:** MIC not detected at the concentration range tested (4–512 μg mL^-1^ for 224C-F2). The upper limit of testing for antibiotics listed in table in parentheses “ND(#)”, and varies by the species-specific parameters for drug resistance.

**—:** Not tested.

### Repeated exposure to 224C-F2 does not lead to resistance

**Fig 6:**
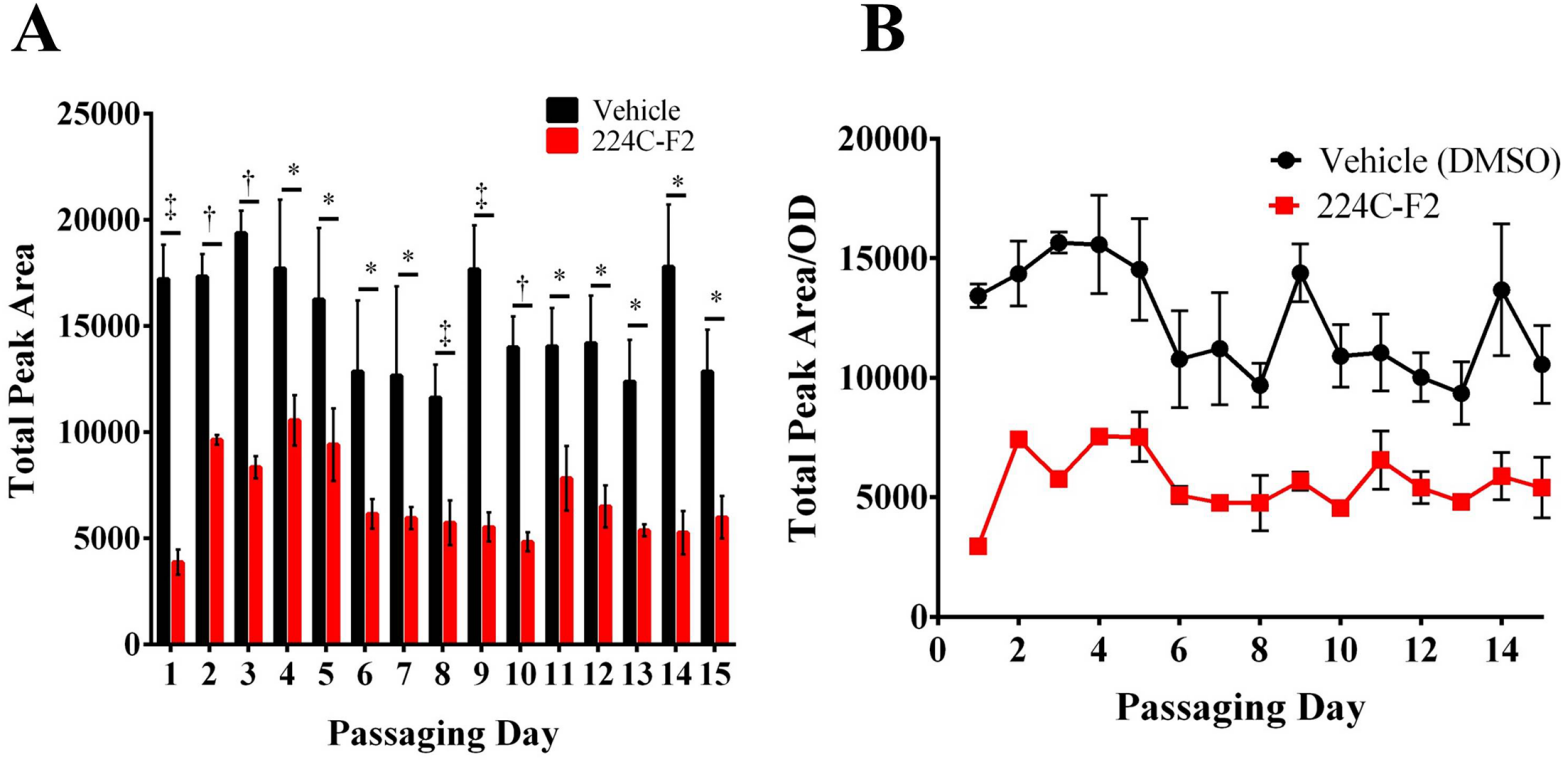
224C-F2 attenuates virulence without any detectable resistance after 15 days of drug passaging. Cultures of USA500 isolate NRS385 (*agr* group I) were passaged for 15 consecutive days in the presence of 16 μg mL^-1^ of 224C-F2. **(A)** The sum total peak area of de-formylated and formylated delta toxin was quantified for the mock vehicle control (DMSO) and treated group. A significant difference (p<0.05) was evident for all treatment days. **(B)** 224C-F2 inhibited delta-toxin production over the length of the passaging experiment in the absence of growth inhibition. Significant differences between treatment and vehicle are represented as: *: *p*<0.05; ‡: *p*<0.01; †: *p*<0.001.

Antibiotic resistance is a major concern in any anti-infective drug discovery initiative. Here, we hypothesized that targeting bacterial virulence with a multi-component botanical therapy—potentially containing multiple actives acting on multiple targets–would not be very likely to generate resistance. As reporter strains can lose their effectiveness in tracking activity over multiple passaging days (e.g. due to loss of the plasmid), we chose to design a new method for tracking the quorum quenching efficacy of our lead composition (224C-F2). This was achieved through use of a high toxin output strain (NRS385) that has been shown to consistently produce high levels of δ-toxin in the supernatant. Bacterial growth was monitored by OD_600_ and δ-toxin was quantified by HPLC. Data for total peak area measured by HPLC (Fig 6A) and area adjusted for slight differences in daily OD (Fig 6B) both reflect significant differences between the levels of δ-toxin produced by the treated versus control cultures for 15 days of passaging. Moreover, no trends in the shift of this observation towards resistance were noted.

### 224C-F2 is nontoxic to HaCaT cells and mouse skin

**Fig 7:**
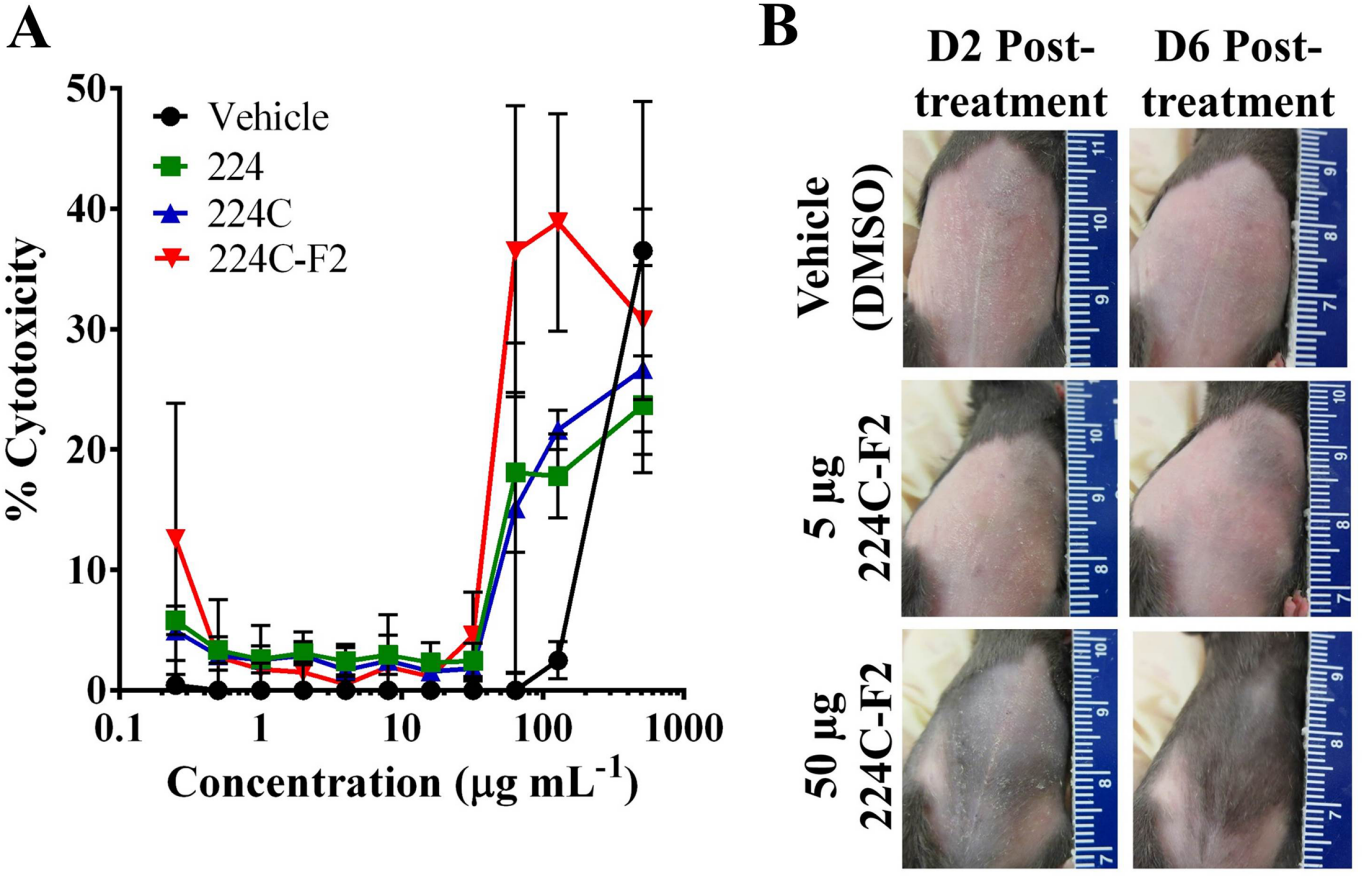
224C-F2 is non-toxic to human keratinocytes and murine skin. **(A)** Immortalized human keratinocytes (HaCaT cells) were treated with up to 512 μg mL^-1^ of extract fractions (24 hrs). The LD_50_ for 224C-F2 could not be determined at this test range, indicating that it is well above the active dose for quorum quenching activity (IC_50_ = 1.56–25 μg mL^-1^, depending on strain). **(B)** Uninfected mice received an intradermal injection of 5 or 50 μg 224C-F2. No gross alterations in skin appearance were observed.

To investigate the potential for cytotoxic or irritant effects of *C*. *sativa* leaf extracts, we treated immortalized human keratinocyte cells with up to 512 μg mL^-1^ of each extract. In all cases (224, 224C, 224C-F2), cytotoxicity (>30%) was only observed at doses at 8–10 times greater than the dose range necessary for quorum quenching activity, and which also corresponded with the rise in toxicity of vehicle treatment alone (DMSO), with no significant difference in cytotoxicity between the vehicle and extracts (Fig 7A). With regards to the potential for irritant or necrotic effects on murine skin, mice were injected intradermally with either 5 μg or 50 μg and monitored for any visible changes in the skin morphology and weight loss. No changes were noted any day at up to 6 days of post-injection follow-up (Fig 7B).

### 224C-F2 attenuates MRSA-induced illness in an *in vivo* skin infection model

**Fig 8:**
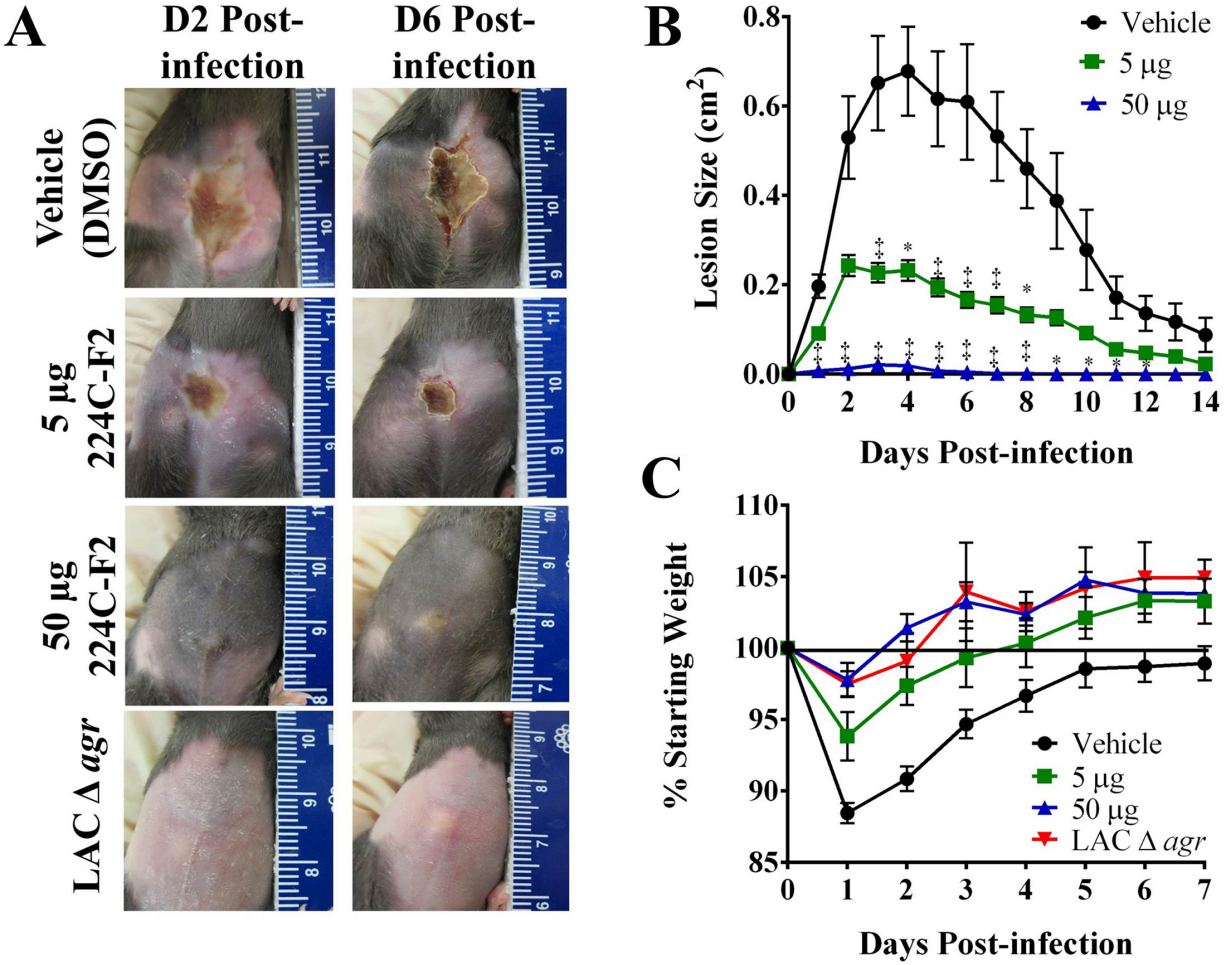
224C-F2 attenuates MRSA-induced dermatopathology in a murine model of skin and soft tissue infection. C5Bl/6 mice were intradermally injected with 1x10^8^ CFUs of LAC (USA 300 isolate, AH1263) or its *agr* deletion mutant (AH1292). Mice received a single dose of 224C-F2 (at 5 or 50 μg) or the vehicle control (DMSO) at the time of infection. Significant differences between treatment and vehicle are represented as: *: p<0.05; ‡: p<0.01. **(A)** Images of abscesses and ulcers on days 2 and 6 post-infection (scale in cm). **(B)** 224C-F2 attenuates dermatopathology with a single dose of either 5 or 50 μg. **(C)** 224C-F2 reduces morbidity and mice do not lose weight.

The *agr* quorum sensing system controls staphylococcal virulence factor expression and is required for necrotic skin lesion formation following cutaneous challenge [29, 35, 37]. Having demonstrated the quorum sensing inhibiting activity of 224C-F2 *in vitro* (Figs 3–6), we next assessed the efficacy of this composition in a mouse model of *S*. *aureus* skin infection. When delivered at the time of infection, 224C-F2 decreased the area of resultant ulcers in a dose-dependent manner (Fig 8A and 8B). In addition, 224C-F2 administration significantly attenuated infection-induced morbidity (assessed by weight loss) compared to vehicle treated controls (Fig 8C). Importantly, mice receiving intradermal injection of 224C-F2 alone did not exhibit any overt signs of dermal irritation or clinical illness *e*.*g*., weight loss, malaise, hunching, coat ruffling (Fig 7B and data not shown). Together these data corroborate the *in vitro* findings and suggest that 224C-F2 impairs MRSA pathogenesis without manifesting local or systemic toxicity.

### Chemical characterization of 224C-F2

**Fig 9:**
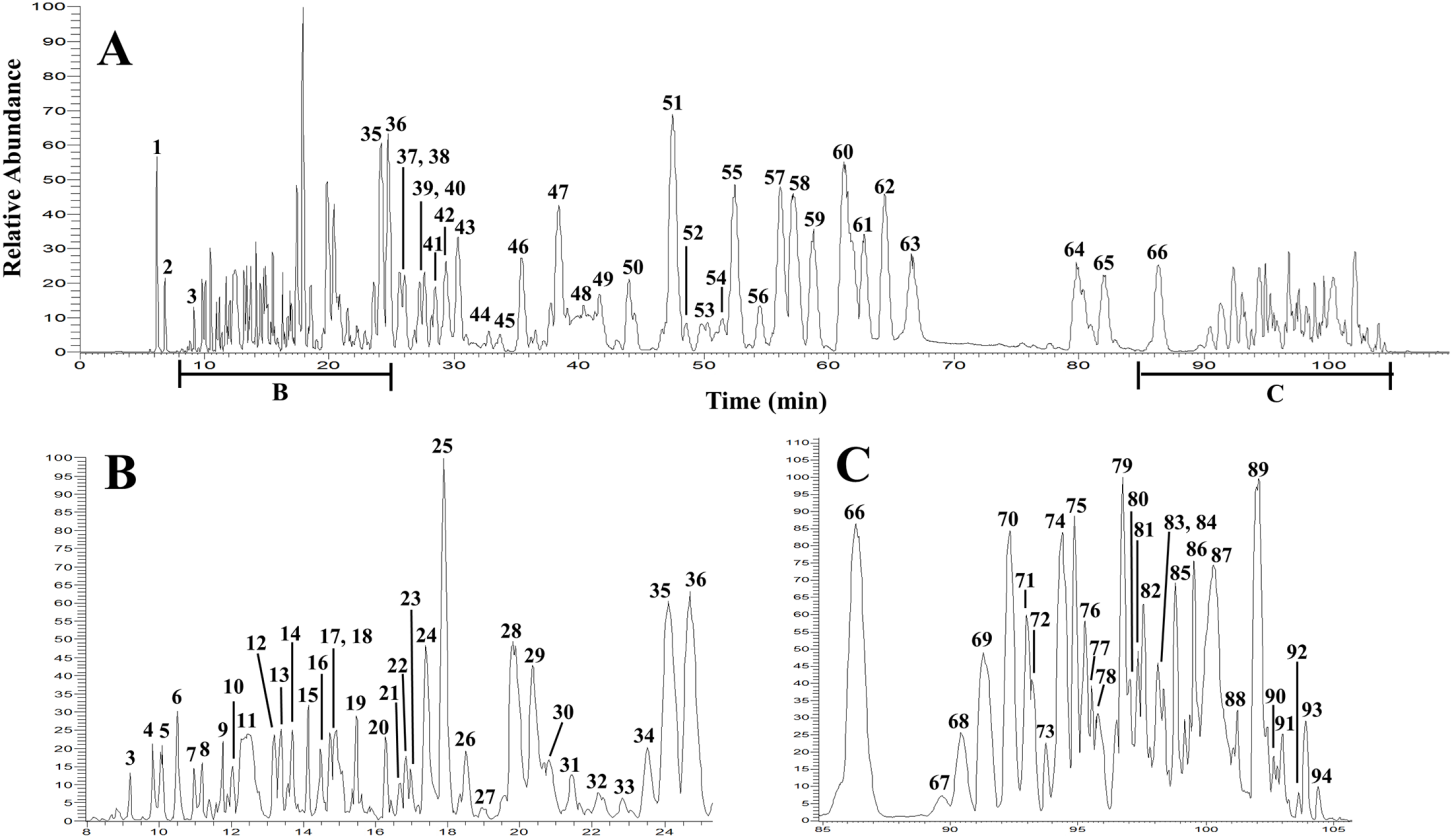
LC-FTMS ESI negative base peak chromatogram for 224C-F2. All peaks correspond to data presented in Table 5. Putative structures are reported in Fig 10.

**Fig 10:**
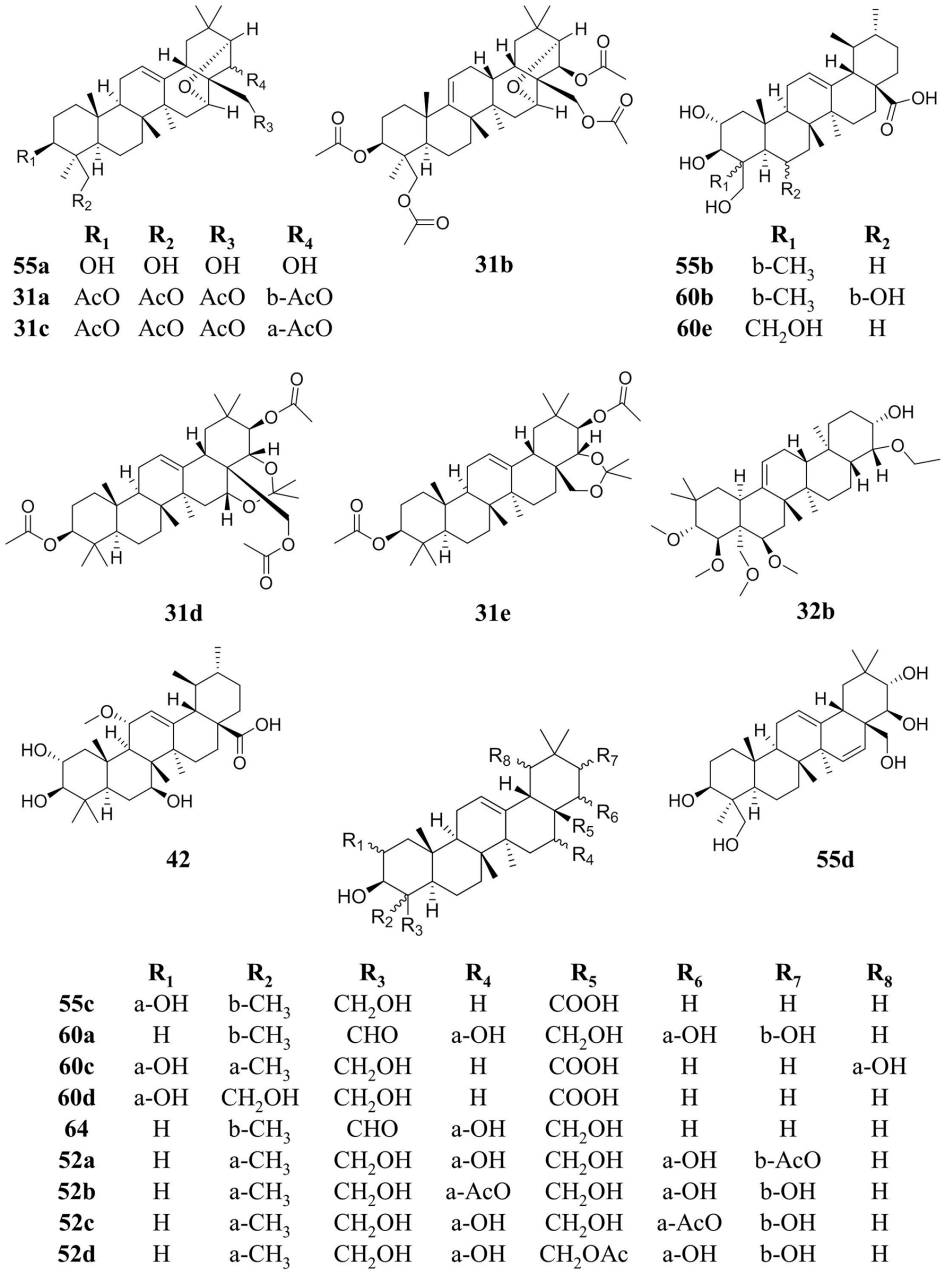
Putative structures of ursene and oleanene derivatives found in the most active region of 224C-F2 (retention time of 21–49 min) were determined following MS analysis and database searches. Compounds are listed by Peak number, corresponding to Table 5. Peak **31** was determined to be C_39_H_59_O_8_ or C_38_H_55_O_9_ with a relative abundance of 0.34%. Putative structural matches include: (**31a**) escigenin tetraacetate (6CI); (**31b**) tetraacetate (7CI, 8CI) 16α, 21α- epoxy-olean- 9(11)—ene- 3β, 22β, 24, 28- tetrol; (**31c**) tetraacetate aescigenin; (**31d**) triacetate (8CI) cyclic 16, 22- acetal-olean- 12- ene- 3β, 16α, 21β, 22α, 28- pentol; (**31e**) triacetate (8CI) cyclic 22, 28- acetal-olean- 12- ene- 3β, 16α, 21β, 22α, 28- pentol. Peak **32** was determined to be C_35_H_59_O_6_ with a relative abundance of 0.30%. Putative structural matches include: (**32a**) stigmastane (Fig 11) and (**32b**) (3β, 4β, 16α, 21β, 22α) -16, 21, 22, 23, 28- pentamethoxy (9CI) olean- 12- en- 3- ol. Peak **42** was determined to be C_31_H_49_O_6_ with a relative abundance of 1.43%. Putative structural matches included (**42**) amirinic acid. Peak **52** was determined to be C_32_H_51_O_7_ with a relative abundance of 0.48%. Putative structural matches include: (**52a**) 21-acetate protoescigenin, (**52b**) 16-acetate protoescigenin, (**52c**) 22-acetate protoescigenin and (**52d**) 28-acetate protoescigenin. Peak **55** was determined to be C_30_H_48_O_5_, with a relative abundance of 4.11%. Putative structural matches include: (**55a**) 16,21-epoxy-(3β,4β,16α,21α,22β)-olean-12-ene-3,22,24,28-tetrol (9CI); (**55b**) asiatic acid; (**55c**) arjunolic acid; (**55d**) isoescigenin. Peak **60** was determined to be C_30_H_48_O_6_, with a relative abundance of 6.80%. Putative structural matches include: (**60a**) camelliagenin E; (**60b**) brahmic acid; (**60c**) sericic acid; (**60d**) belleric acid; and (**60e**) 2,3,23,24-tetrahydroxy-(2α,3β)-urs-12-en-28-oic acid. Peak **64** was determined to be C_30_H_45_O_5_, with a relative abundance of 2.91%. The putative structural match is (**64**) ouillaic acid.

**Fig 11:**
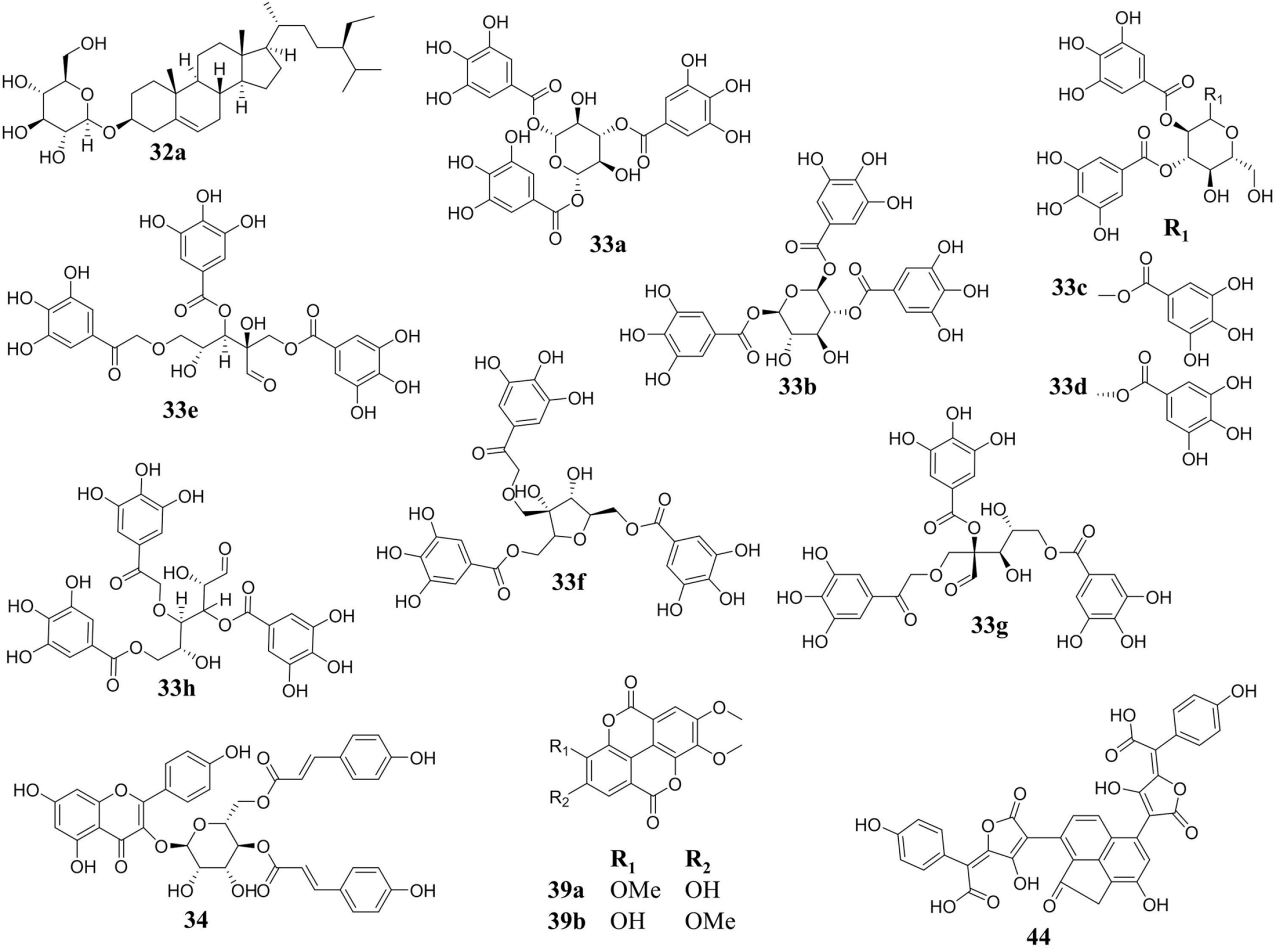
Putative structures of compounds other than pentacyclic triterpenes found in the most active region of 224C-F2 (retention time of 21–49 min). Compounds are listed by Peak number, corresponding to Table 5. Peak **32** was determined to be C_35_H_59_O_6_ with a relative abundance of 0.30%. Putative structural matches include: (**32a**) stigmastane and (**32b**) (3β, 4β, 16α, 21β, 22α) -16, 21, 22, 23, 28- pentamethoxy (9CI) olean- 12- en- 3- ol (Fig 10). Peak **33** was determined to be C_27_H_23_O_18_ with a relative abundance of 0.16%. Putative structural matches include: (**33a**) 1,3,6-tri-O-galloylglucose; (**33b**) 1,2,6-tri-galloyl-β-D-glucose; (**33c**) 1,2,3-tri-O-galloylglucose; (**33d**) 1,2,3-tri-O-galloyl-β-D-glucopyranose; (**33e**) 2',3,5-tri-O-galloyl-D-hamamelose; (**33f**) 2- *C*- [[(3, 4, 5- trihydroxybenzoyl) oxy] methyl]- 1, 5- bis(3, 4, 5- trihydroxybenzoate) D- Ribofuranose; (**33g**) kurigalin; (**33h**) 3,4,6-tri-O-galloyl-D-glucose. Peak **34** was determined to be C_39_H_31_O_15_ with a relative abundance of 0.65%. Putative structural matches include: (**34**) castanoside B. Peak **39** was determined to be C_17_H_11_O_8_ or C_20_H_11_O_4_N_2_ with a relative abundance of 0.72%. Putative structural matches include: (**39a**) 3,4,3'-tri-O-methylellagic acid and (**39b**) 3,3',4'-tri-O-methylellagic acid. Peak **44** was determined to be C_34_H_29_O_15_ with a relative abundance of 0.26%. Putative structural matches included (**44**) norbadione A.

The percent yield of extract from the dry leaves was 43.98% for extract 224, 2.716% for 224C and 1.155% for 224C-F2 (Fig 2). LC-FTMS analysis of 224C-F2 revealed the presence of at least 94 compounds (Table 5). The greatest quorum quenching effects of 224C-F2 were observed in the retention time region of 21–49 min (Fig 9), suggesting the presence of several distinct quorum quenching compounds (data not shown). Specifically, there are 22 compounds found in this region, 10 present at >1% relative abundance. These correspond to peak numbers, predicted formulas, and relative abundances of: **35** C_57_H_24_O_2_ (2.67%), **36** C_27_H_50_O_6_ (2.65%), **42** C_31_H_50_O_6_ (1.43%), **43** C_30_H_46_O_7_ (1.86%), **46** and **47** C_57_H_23_O_2_N_3_ (1.64% and 3.13%, respectively), **48** and **49** C_59_H_25_O_3_ (1.45 and 1.07%, respectively), **50** C_41_H_33_O_16_ (1.20%) and **51** C_30_H_47_O_5_ (5.96%). Putative structures for 7 peaks were determined to be pentacyclic triterpenes (specifically, oleanene and ursene derivatives) based on accurate mass analysis, fragmentation patterns, and comparison with natural product databases (Fig 10), and these collectively represent 16.37% in relative abundance. Of note, while present at relative abundance levels of <1% each, the putative structures of gallotannins (**32**, **33**, **34**) and ellagitannins (**39**) were also identified in the most active region of 224C-F2 (Fig 11).

**Table 5:** Mass spectrometry (m/z) analysis of 224C-F2. The corresponding chromatogram is reported in Fig 9; putative structures in Figs 10 and 11.

Peak No.	Retention Time (min)	Relative Abundance	Formula (Δ ppm)	m/z*([M-H]^-^ in bold)	MS^2^	UV
**1**	6.21	0.99	C_14_H_9_O_10_ (0.950)	**337.02044**, 675.05002	293.2077	220, 270
**2**	6.85	0.39	C_14_H_11_O_10_ (0.586)	339.0354	169.104	220, 270
**3**	9.17	0.12	C_14_H_9_O_8_ (1.993)	**305.03035**, 611.06804	179.1553	ND
**4**	9.82	0.24	C_14_H_9_O_9_ (2.347)	**321.02541**, 643.05813	169.1691	215, 280
**5**	10.05	0.32	C_31_H_17_O_15_N (0.313)	321.02581, 579.15259, 611.10667, **643.06011**	599.13610, 626.17633	215, 280
**6**	10.49	0.36	C_8_H_7_O_5_ (2.031)	**183.02972**, 367.06658	124.08116, 168.05430, 183.09955	215, 275
**7**	10.94	0.14	C_14_H_9_O_9_ (2.097)	**321.02533**, 643.05801	169.1772	ND
**8**	11.18	0.16	C_18_H_15_O_8_ (2.138)	33.06169, **359.07746**	179.1279	215, 325
**9**	11.74	0.22	C_21_H_25_O_8_ (1.898)	**405.15626**, 451.23452, 521.24023	225.224	ND
**10**	12.02	0.19	C_25_H_39_O_7_ (1.337)	269.13963, **451.27073**	407.24461, 433.23288	ND
**11**	12.39	1.24	C_28_H_11_O_16_ (2.757)	603.0069	465.2773	255, 365
**12**	13.18	0.33	C_15_H_11_O_9_ (1.537)	**335.04137**, 671.08973	183.209	ND
**13**	13.36	0.32	C_29_H_33_O_10_ (2.420)	421.15146, **541.20923**	491.18281, 523.30375	ND
**14**	13.68	0.33	C_15_H_11_O_9_ (1.358)	**335.04131**, 671.09010	183.1594	215, 280, 305 (s)
**15**	14.13	0.33	C_12_H_19_O_5_ (1.329)	**243.12412**, 487.25602	225.1935	ND
**16**	14.47	0.30	C_12_H_19_O_5_ (1.288)	**243.12411**, 487.25527	181.17663, 183.17220, 199.20072, 225.24659	ND
**17**	14.71	0.26	C_23_H_23_O_5_N_4_ (-0.076)	435.1674	259.15392, 388.97603	ND
**18**	14.90	0.54	C_33_H_13_O_13_N (0.081)	391.14110, **631.03874**	479.4478	220, 255 (s), 320
**19**	15.44	0.32	C_20_H_31_O_8_ (2.101)	399.2033	381.33071, 355.30539, 337.33447	ND
**20**	16.26	0.25	C_30_H_39_O_8_ (2.046)	527.2661	263.20418, 459.45849	ND
**21**	16.63	0.16	C_59_H_41_O (-1.084)	405.15614, **765.31542**	613.37801, 617.27520	ND
**22**	16.84	0.21	C_25_H_23_O_13_ (1.179)	599.1065	255.10369, 284.12921, 285.12352, 327.22129, 471.26204	ND
**23**	16.97	0.17	C_15_H_9_O_6_ (0.873)	**285.04071**, 571.08859	175.11613, 199.13418, 241.14281	ND
**24**	17.39	1.15	C_15_H_9_O_7_ (1.907)	**301.03595**, 603.08092, 905.12920	151.08622, 179.07130	215, 255, 370
**25**	17.88	2.08	C_18_H_31_O_5_ (1.414)	**327.21816**, 655.44500	211.20899, 229.25489, 291.32982	220, 280, 330
**26**	18.50	0.40	C_26_H_39_O_6_ (1.918)	**447.27607**, 493.28185, 895.56483	367.44245, 385.41613, 401.41446, 429.40811	ND
**27**	18.97	0.08	C_25_H_23_O_13_ (1.461)	519.33352, 531.11519, 564.33918	471.23936, 489.30998	ND
**28**	19.81	1.90	C_43_H_65_O_24_ (-0.265)	329.23393, 635.14327, **965.38687**	635.3972	220, 270, 315
**29**	20.33	1.31	C_50_H_19_O (-1.840)	635.143	285.15142, 489.24712, 575.28563	ND
**30**	20.79	0.50	C_18_H_31_O_5_ (1.842)	**327.21830**, 655.44361	171.19486, 309.30290	ND
**31**	21.45	0.34	C_39_H_59_O_8_ (34.188); C_38_H_55_O_9_ (89.700)	327.21808, 635.14199, **655.44397**	611.57685, 637.50026	220, 345
**32**	22.15	0.30	C_35_H_59_O (39.112)	287.22306, 327.21853, 419.16528, **575.45419**, 661.36183, 755.16549	515.51088, 531.44821, 557.36796	220, 365
**33**	22.76	0.16	C_27_H_23_O_18_ (80.837)	327.21797, **635.1404**	285.14949, 489.26353	220, 315
**34**	23.54	0.65	C_39_H_31_O_15_ (1.118)	739.1677	285.14592, 453.23143	220, 270, 315
**35**	24.11	2.67	C_57_H_23_O_2_ (-1.263)	**739.16945**, 785.17519	285.13965, 453.25508, 575.28574, 593.30977	220, 315
**36**	24.70	2.65	C_27_H_41_O_6_ (2.250)	**461.29190**, 507.29838, 923.59481	399.45155, 415.44332, 443.44095	ND
**37**	25.61	0.90	C_57_H_23_O_2_ (-1.520)	**739.16923**, 785.17625	285.14029, 453.24568, 575.300080, 593.27713	220, 315
**38**	26.00	0.84	C_55_H_21_ON_3_ (-0.163)	**739.16895**, 785.17576	285.14254, 453.25317, 575.30248, 593.29857	220, 315
**39**	27.18	0.72	C_17_H_11_O_8_ (3.317); C_20_H_11_O_4_N_2_ (75.038)	**343.04651**, 687.10014	328.2618	225, 370
**40**	27.57	0.84	C_40_H_27_O_11_N_4_ (0.026)	739.1683	285.15812, 453.24020, 575.26598, 593.28341	ND
**41**	28.45	0.68	C_34_H_29_O_15_ (0.822)	**677.15166**, 723.15962	284.13571, 557.27766, 617.28692	220, 310
**42**	29.30	1.43	C_31_H_49_O_6_ (-68.377)	**517.31804**, 563.32400, 723.15771, 797.17555, 1035.64788	437.46551, 455.47822	ND
**43**	30.28	1.86	C_30_H_45_O_7_ (2.500)	**517.31837**, 563.32368, 1035.64687	437.46548, 455.47972, 499.50877	ND
**44**	32.74	0.26	C_34_H_29_O_15_ (-1.423)	677.1506	285.14940, 531.27599, 617.30259	ND
**45**	33.59	0.31	C_34_H_29_O_15_ (-1.112)	547.32815, **677.15045**	285.1559, 531.29170	ND
**46**	35.37	1.64	C_57_H_23_O_2_N_3_ (-0.557)	**781.17897**, 827.18606	285.14510, 495.29220, 617.28374, 635.31959	225, 310
**47**	38.38	3.13	C_57_H_23_O_2_N_3_ (0.288)	**781.17980**, 827.18720	285.14324, 495.26366, 635.32730	220, 315
**48**	40.35	1.45	C_59_H_25_O_3_ (-0.676)	445.29698, 491.30248, **781.18038**, 827.18746, 1227.49773	285.14614, 496.26557, 635.31187	220, 285, 310
**49**	41.60	1.07	C_59_H_25_O_3_ (-1.201)	533.34940, **781.18002**, 827.18679	285.12660, 495.27804, 635.31517	220, 305
**50**	43.97	1.20	C_41_H_33_O_16_ (0.617)	781.1779	285.13183, 495.28281, 635.30008	220, 295
**51**	47.42	5.96	C_30_H_47_O_5_ (2.221)	**487.34398**, 533.35098, 975.70011	469.4979	ND
**52**	48.55	0.48	C_32_H_51_O_7_ (-0.543)	**547.36365**, 593.36950	529.51760, 529.51407	ND
**53**	49.73	0.93	C_31_H_49_O_7_ (-1.063)	533.3478	435.49468, 486.52688, 515.46714	ND
**54**	51.40	0.67	C_30_H_47_O_5_ (-0.508)	**487.34265**, 533.34807	485.22061, 486.02828, 487.97113	ND
**55**	52.42	4.11	C_30_H_47_O_5_ (2.262)	**487.34400**, 533.35101, 975.70046	469.499	225, 270
**56**	54.43	0.97	C_31_H_49_O_8_ (2.418)	531.33301, **549.34462**	489.57421, 531.41583	ND
**57**	56.01	3.84	C_30_H_47_O_5_ (2.385)	**487.34406**, 533.35100, 975.70018	441.51916, 469.49007	ND
**58**	57.15	4.36	C_59_H_25_O_3_N_3_ (-0.498)	**823.18973**, 869.19736	285.13804, 677.32656	225, 315
**59**	58.72	2.88	C_59_H_25_O_3_N_3_ (-0.668)	**823.18959**, 869.19629	285.14787, 677.31854	220, 315
**60**	61.17	6.80	C_30_H_47_O_6_ (2.359)	**503.33900**, 549.34625, 1007.69059	471.4702	ND
**61**	62.79	2.56	C_59_H_25_O_3_N_3_ (-0.790)	**823.18949**, 869.19646	285.13196, 677.32432	220, 315
**62**	64.42	3.63	C_59_H_25_O_3_N_3_ (-1.020)	823.1893	285.12941, 677.31768	220, 315
**63**	66.55	3.09	C_30_H_47_O_6_ (1.783)	**503.33871**, 549.34568, 1007.69071	319.32486, 401.40810, 471.48019	ND
**64**	79.78	2.91	C_30_H_45_O_5_ (2.930)	**485.32867**, 531.33501, 971.66969	423.49110, 467.49405	ND
**65**	81.93	1.98	C_30_H_49_O_5_ (1.517)	**489.35929**, 535.36558, 979.72844	471.5105	ND
**66**	86.29	2.25	C_27_H_41_O_5_ (1.151)	**445.29464**, 491.30202, 891.60136	383.45557, 427.42744	ND
**67**	89.69	0.13	C_31_H_49_O_7_ (-0.744)	533.348	487.4407	ND
**68**	90.42	0.46	C_31_H_51_O_6_ (-1.179)	519.3685	415.49682, 487.52239	ND
**69**	91.30	0.92	C_39_H_53_O_7_ (0.273)	**633.37985**, 679.38594	179.08913, 454.54671, 590.61801	225, 295, 305
**70**	92.33	1.20	C_32_H_49_O_6_ (1.998)	**529.35452**, 575.36114, 1059.72276	469.5822	ND
**71**	92.99	0.51	C_32_H_49_O_6_ (2.281)	**529.35467**, 575.36044	469.6057	ND
**72**	93.19	0.31	C_27_H_43_O_4_ (3.842)	**431.31834**, 477.32329, 529.35529	ND	ND
**73**	93.74	0.16	C_40_H_55_O_9_ (0.447)	547.36537, **679.38546**	619.46845, 661.52463	ND
**74**	94.38	1.26	C_53_H_99_O_13_ (1.032)	471.35048, 517.35526, **943.71008**	471.52443, 925.86052	ND
**75**	94.85	0.69	C_39_H_53_O_7_ (2.957)	633.3816	470.54105, 514.52351, 590.59043	ND
**76**	95.27	0.47	C_32_H_49_O_6_ (2.583)	**529.35483**, 575.36059	469.5901	ND
**77**	95.52	0.18	C_32_H_49_O_7_ (2.022)	**545.34949**, 591.35525	485.4473	ND
**78**	95.80	0.40	C_33_H_45_ON (-1.238)	**471.35008**, 517.35387, 943.70544	ND	ND
**79**	96.74	0.82	C_32_H_49_O_7_ (2.169)	**545.34956**, 591.35536	453.49263, 485.45412, 513.44457	ND
**80**	97.00	0.25	C_31_H_49_O_7_ (1.487)	485.32855, **533.34917**, 591.35511	489.44583, 513.21950	ND
**81**	97.34	0.29	C_32_H_49_O_7_ (1.564)	**545.34923**, 591.35526	485.4417	ND
**82**	97.56	0.44	C_33_H_45_ON (-3.677)	**471.34893**, 517.35439	453.5104	ND
**83**	98.10	0.39	C_48_H_59_O_10_ (-0.982)	795.4106	633.6017	225, 300, 325
**84**	98.33	0.23	C_48_H_59_O_10_ (0.351)	485.32931, 531.33456, **795.41162**	633.5975	ND
**85**	98.81	0.56	C_20_H_39_O_7_ (3.152)	391.2714	371.2172	ND
**86**	99.53	0.57	C_39_H_57_O_6_ (1.685)	475.30817, 533.34947, **621.41711**	179.11584, 451.48783, 577.68246, 603.59836	ND
**87**	100.29	1.85	C_27_H_41_O_4_ (1.717)	**429.30177**, 475.30774	367.42174, 411.43779	ND
**88**	101.24	0.21	C_30_H_49_O_4_ (2.190)	**473.36460**, 519.36979	ND	ND
**89**	102.05	1.32	C_30_H_47_O_4_ (2.306)	**471.34907**, 517.35508, 943.70971	367.41415, 409.51672, 453.51813	ND
**90**	102.67	0.10	C_39_H_53_O_6_ (0.028)	455.31789, 501.32245, **617.38478**	497.49844, 573.61572	ND
**91**	103.00	0.17	C_30_H_47_O_5_ (0.394)	**487.34309**, 533.34826, 975.69540	469.4995	ND
**92**	103.59	0.04	C_29_H_45_O_4_ (0.452)	**457.33254**, 503.33759, 915.67143	395.50910, 439.45901	ND
**93**	103.90	0.20	C_30_H_47_O_4_ (1.075)	**471.34849**, 517.35330, 943.70324	413.50334, 453.50566	ND
**94**	104.39	0.06	C_30_H_47_O_5_ (0.169)	**487.34298**, 533.34853, 975.69146	455.4734	225, 290, 435

*m/z: When multiple base ions were formed, the number in **bold font** indicates the ion that was used to predict empirical formula and underwent MS^2^ fragmentation.

ND = not detected.

224C-F2 was also examined by HPLC-DAD and LC-FTMS for the presence of 5 compounds reported to be found in crude *C*. *sativa* leaf extracts [42], and it was determined that 224C-F2 does not contain chlorogenic acid, ellagic acid, hyperoside, isoquercitrin, or rutin.

## Discussion

The ethnobotanical approach to drug discovery [55] was used here to identify *Castanea sativa* leaves as a potential source new anti-infective agents. Through design of a bioactivity-guided fractionation strategy based on limited growth-impact coupled to quorum sensing inhibition, we were successful in creating a highly efficacious botanical composition with universal quenching activity for all *agr* alleles. To the best of our knowledge, the present work represents the first in-depth investigation of European Chestnut leaf extract for its quorum quenching and anti-virulence effects since its identification as a potential quorum quenching lead [4]. Furthermore, this is the first report of the quorum quenching effects of a botanical composition rich in ursene and oleanene derivatives (Fig 10) against *S*. *aureus*. Additional compounds identified in the most active region (at <1% relative abundance each) included putative gallotannins, which share a tri-galloyl structure with varying core sugars (**32**, **33**, **34**), and a putative ellagitannin (**39**). It is possible that in addition to the pentacyclic triterpenes present in 224C-F2, hydrolysable tannins also contribute to the extract’s quorum quenching activity.

European Chestnut leaf extracts have been the focus of a number of studies centered on evaluation of its activity in scavenging reactive oxygen species [42, 56] and cytoprotective effects, specifically with regards to protection from UV-damage in skin cells [57]. The examination of European Chestnut leaf extracts with a patch test revealed that with respect to irritant effects, such extracts can be considered as safe for topical applications [58]. The integration of *C*. *sativa* leaf extracts into cosmetic compositions has also been patented, and is based on the antibacterial and reactive oxygen species (ROS) scavenging effects of the extract [59]. Our safety studies in both human keratocytes (HaCaT cells) and murine skin (Fig 6) have reconfirmed that this version of European Chestnut leaf extract (224C-F2) can be considered safe for topical applications based on its lack of cytotoxic and irritant effects.

Several layers of evidence in support of the efficacy of *C*. *sativa* leaf extracts in blocking *S*. *aureus* virulence have been presented. Specifically, we have demonstrated that European Chestnut leaf extracts are effective in blocking production of the translational products of RNAIII, including a number of exotoxins. Overall virulence was quenched as demonstrated by the lack of cytotoxic effects elicited by supernatants of cultures treated with the extract. Importantly, using an *in vivo* model, we have demonstrated efficacy in attenuating dermonecrosis, even in the absence of adjuvant antibiotics.

This inhibition of virulence and pathogenesis was accomplished without posing growth inhibitory pressures on not only *S*. *aureus*, but also a panel of common members of the human cutaneous microbiome. A robust skin microflora is critical to skin barrier health and prevention of disease onset. The majority of the bacterial cutaneous microbiome is represented by Actinobacteria, Firmicutes, Proteobacteria and Bacteroidetes [60]. Much like cases of dysbiosis in gut microflora, broad-spectrum activity against the skin microflora also holds the potential for fostering an environment amenable to the proliferation of pathogenic bacteria [61]. The presence of commensals, like *Staphylococcus epidermidis*, is essential to state of host innate immunity [62]. Thus, it is noteworthy that 224C-F2 specifically blocks *S*. *aureus* virulence without adding selective pressures on major representatives of the cutaneous microbiome.

The mechanistic basis for 224C-F2’s quorum quenching activity remain unclear. Multiple lines of evidence suggest that components within 224C-F2 directly target the core machinery of the *agr* system, such as our observation of *agr* P3 promoter reduction (Fig 3) and reduced levels of δ-toxin production (Fig 4), which is encoded within RNAIII transcript regulated by P3. If 224C-F2 only targeted downstream factors regulated by quorum sensing, such as α-hemolysin, inhibition of *agr* P3 or δ-toxin production would not have been expected. Potential targets within the *agr* system include inhibition of AIP docking with AgrC, prevention of AIP production through AgrB, or reduction of AgrA activation (Fig 1). Future studies will seek to resolve the mechanism, and this will be facilitated by the isolation of individual active components for incorporation in structure-activity relationship (SAR) studies.

We hypothesized that use of a complex mixture that targets an indirect pathway to pathogen success (rather than direct targeting for growth and survival) would be unlikely to result the generation of resistant mutations. In fact, following 15 days of sequential passaging with 224C-F2 *in vitro*, no resistance was detected. This is not surprising; recent findings comparing individual natural products to complex botanical compositions in other targets, such as multidrug-resistant malaria, have demonstrated that single-compound drugs may not be the best answer. For example, in the face of growing artemisinin resistance for malaria, more chemically complex whole plant therapies (*Artemisia annua* L., Asteraceae) have demonstrated superior efficacy to the single compound in preventing drug resistance [63]. Indeed, complex botanical compositions that meet the FDA standards for safety and efficacy are eligible for an alternative regulatory approval pathway as “botanical drugs”, which are distinct from dietary supplements, and are standardized to levels of marker compounds and regulated like other single compound pharmaceuticals once approved [64]. Two examples of successful botanical drugs include Veregen (*Camellia sinensis* (L.) Kuntze, Theaceae, sinecatechin topical formulation for anogenital warts) and Fulyzaq (*Croton lechleri* Müll. Arg., Euphorbiaceae, procyanidin and prodelphinidin oral formulation for HIV/AIDS-related diarrhea).

While it is debatable whether virulence inhibitors will ever serve as stand-alone therapeutics, many agree that their application as adjuvants to existing lines of antibiotics could be a critical tool in this era of rising antibiotic resistance. Specifically, by inhibiting *agr*, such a therapy effectively blocks the production of an entire suite of diverse staphylococcal toxins, ranging from immune-attacking PSMs, pore-forming hemolysins, and a number of other proteases and lipases that damage the host tissue and weaken the host immune response. This will be of particular relevance to patients faced with toxin-mediated infection, including staphylococcal scalded skin syndrome (esp. in neonates), abscesses, necrotizing fasciitis, sepsis, atopic dermatitis (eczema) and more.

In conclusion, we have demonstrated that a folk-medical treatment for skin inflammation and SSTIs that does not demonstrate “typical” antibacterial activity (bacteriostatic or bactericidal) nevertheless shows great potential for development as a therapeutic due to its ability to specifically target and quench *S*. *aureus* virulence. The results of this study are important not only to future antibiotic discovery and development efforts, but are also vital to the validation of this previously poorly understood traditional medicine as an efficacious therapy, and not simply an unsubstantiated relict of folklore. Importantly, this composition was non-toxic to human keratinocytes and no dermatopathology was noted upon administration to murine skin. Moreover, the composition did not inhibit growth of the normal skin microflora, suggesting that its disruptive action on the cutaneous microbiome would be minimal to nil. Future work will focus on evaluation of individual actives within the composition with the aim of determining whether a complex mixture, such as 224C-F2 or a single compound will prove most effective against all *agr* alleles and which will be least likely to develop resistance when administered under multiple selective pressures, such as for *in vivo* administration as an antibiotic adjuvant.

## References

[1] CDC. Antibiotic resistance threats in the United States, 2013. Atlanta, GA: Centers for Disease Control, 2013.

[2] Ross-Gillespie A, Kümmerli R. Collective decision-making in microbes. Front Microbiol. 2014;5 10.3389/fmicb.2014.00054 24624121 PMC3939447

[3] Ling LL, Schneider T, Peoples AJ, Spoering AL, Engels I, Conlon BP, et al A new antibiotic kills pathogens without detectable resistance. Nature. 2015;517(455–459). 10.1038/nature14098 PMC7414797 25561178

[4] Quave CL, Plano LRW, Bennett BC. Quorum sensing inhibitors of *Staphylococcus aureus* from Italian medicinal plants. Planta Med. 2011;77(02):188–95. 10.1055/s-0030-1250145 20645243 PMC3022964

[5] Quave C, Plano L, Pantuso T, Bennett B. Effects of extracts from Italian medicinal plants on planktonic growth, biofilm formation and adherence of methicillin-resistant *Staphylococcus aureus* . J Ethnopharmacol. 2008;118(3):418–28. 10.1016/j.jep.2008.05.005 18556162 PMC2553885

[6] Quave CL, Estévez-Carmona M, Compadre CM, Hobby G, Hendrickson H, Beenken KE, et al Ellagic acid derivatives from *Rubus ulmifolius* inhibit *Staphylococcus aureus* biofilm formation and improve response to antibiotics. PLoS One. 2012;7(1):e28737 10.1371/journal.pone.0028737 22242149 PMC3252291

[7] Pieroni A, Quave CL, Villanelli ML, Mangino P, Sabbatini G, Santini L, et al Ethnopharmacognostic survey on the natural ingredients used in folk cosmetics, cosmeceuticals and remedies for healing skin diseases in the inland Marches, Central-Eastern Italy. J Ethnopharmacol. 2004;91(2–3):331–44. 10.1016/j.jep.2004.01.015 15120458

[8] Kluytmans J, van Belkum A, Verbrugh H. Nasal carriage of Staphylococcus aureus: epidemiology, underlying mechanisms, and associated risks. Clin Microbiol Rev. 1997;10(3):505–20. 9227864 10.1128/cmr.10.3.505 PMC172932

[9] Novick RP. Autoinduction and signal transduction in the regulation of staphylococcal virulence. Mol Microbiol. 2003;48(6):1429–49. 12791129 10.1046/j.1365-2958.2003.03526.x

[10] Tsuji BT, Rybak MJ, Cheung CM, Amjad M, Kaatz GW. Community- and health care-associated methicillin-resistant *Staphylococcus aureus*: a comparison of molecular epidemiology and antimicrobial activities of various agents. Diagn Microbiol Infect Dis. 2007;58(1):41–7. 17300912 10.1016/j.diagmicrobio.2006.10.021

[11] Otto M. *Staphylococcus aureus* toxins. Curr Opin Microbiol. 2014;17(0):32–7. 10.1016/j.mib.2013.11.004 24581690 PMC3942668

[12] Foster TJ, Geoghegan JA, Ganesh VK, Hook M. Adhesion, invasion and evasion: the many functions of the surface proteins of *Staphylococcus aureus* . Nat Rev Microbiol. 2014;12(1):49–62. 10.1038/nrmicro3161 24336184 PMC5708296

[13] Spaan AN, Surewaard BGJ, Nijland R, van Strijp JAG. Neutrophils versus *Staphylococcus aureus*: A biological tug of war. Annu Rev Microbiol. 2013;67(1):629–50. 10.1146/annurev-micro-092412-155746 23834243

[14] Thoendel M, Kavanaugh JS, Flack CE, Horswill AR. Peptide signaling in the staphylococci. Chem Rev. 2010;111(1):117–51. 10.1021/cr100370n 21174435 PMC3086461

[15] Zhu J, Kaufmann GF. Quo vadis quorum quenching? Curr Opin Pharmacol. 2013;13(5):688–98. 10.1016/j.coph.2013.07.003 23876839

[16] Quave CL, Horswill AR. Flipping the switch: Tools for detecting small molecule inhibitors of staphylococcal virulence. Frontiers in Microbiology. 2014;5 10.3389/fmicb.2014.00706 25566220 PMC4264471

[17] Hersh AL, Chambers HF, Maselli JH, Gonzales R. National trends in ambulatory visits and antibiotic prescribing for skin and soft-tissue infections. Arch Intern Med. 2008;168(14):1585–91. 10.1001/archinte.168.14.1585 18663172

[18] Daum RS. Clinical practice. Skin and soft-tissue infections caused by methicillin-resistant *Staphylococcus aureus* . N Engl J Med. 2007;357(4):380–90. 17652653 10.1056/NEJMcp070747

[19] Stryjewski ME, Chambers HF. Skin and soft-tissue infections caused by community-acquired methicillin-resistant *Staphylococcus aureus* . Clin Infect Dis. 2008;46 Suppl 5:S368–77. 10.1086/533593 18462092

[20] Wright JS 3rd, Jin R, Novick RP. Transient interference with staphylococcal quorum sensing blocks abscess formation. Proc Natl Acad Sci USA. 2005;102(5):1691–6. 15665088 10.1073/pnas.0407661102 PMC547845

[21] Park J, Jagasia R, Kaufmann GF, Mathison JC, Ruiz DI, Moss JA, et al Infection control by antibody disruption of bacterial quorum sensing signaling. Chem Biol. 2007;14(10):1119–27. 17961824 10.1016/j.chembiol.2007.08.013 PMC2088803

[22] Schwan WR, Langhorne MH, Ritchie HD, Stover CK. Loss of hemolysin expression in *Staphylococcus aureus agr* mutants correlates with selective survival during mixed infections in murine abscesses and wounds. FEMS Immunol Med Microbiol. 2003;38(1):23–8. 12900051 10.1016/S0928-8244(03)00098-1

[23] Mayville P, Ji G, Beavis R, Yang H, Goger M, Novick RP, et al Structure-activity analysis of synthetic autoinducing thiolactone peptides from *Staphylococcus aureus* responsible for virulence. Proc Natl Acad Sci USA. 1999;96(4):1218–23. 9990004 10.1073/pnas.96.4.1218 PMC15443

[24] Wright JD, Holland KT. The effect of cell density and specific growth rate on accessory gene regulator and toxic shock syndrome toxin-1 gene expression in *Staphylococcus aureus* . FEMS Microbiol Lett. 2003;218:377–83. 12586420 10.1016/S0378-1097(02)01193-X

[25] Kennedy AD, Wardenburg JB, Gardner DJ, Long D, Whitney AR, Braughton KR, et al Targeting of alpha-hemolysin by active or passive immunization decreases severity of USA300 skin infection in a mouse model. J Infect Dis. 2010;202(7):1050–8. 10.1086/656043 20726702 PMC2945289

[26] Heyer G, Saba S, Adamo R, Rush W, Soong G, Cheung A, et al *Staphylococcus aureus agr* and *sarA* functions are required for invasive infection but not inflammatory responses in the lung. Infect Immun. 2002;70(1):127–33. 11748173 10.1128/IAI.70.1.127-133.2002 PMC127645

[27] Bubeck Wardenburg J, Patel RJ, Schneewind O. Surface proteins and exotoxins are required for the pathogenesis of *Staphylococcus aureus* pneumonia. Infect Immun. 2007;75(2):1040–4. 17101657 10.1128/IAI.01313-06 PMC1828520

[28] Bubeck Wardenburg J, Bae T, Otto M, Deleo FR, Schneewind O. Poring over pores: alpha-hemolysin and Panton-Valentine leukocidin in *Staphylococcus aureus* pneumonia. Nat Med. 2007;13(12):1405–6. 18064027 10.1038/nm1207-1405

[29] Montgomery CP, Boyle-Vavra S, Daum RS. Importance of the global regulators Agr and SaeRS in the pathogenesis of CA-MRSA USA300 infection. PLoS One. 2010;5(12):e15177 10.1371/journal.pone.0015177 21151999 PMC2996312

[30] Kennedy AD, Otto M, Braughton KR, Whitney AR, Chen L, Mathema B, et al Epidemic community-associated methicillin-resistant *Staphylococcus aureus*: Recent clonal expansion and diversification. Proc Natl Acad Sci USA. 2008;105(4):1327–32. 10.1073/pnas.0710217105 18216255 PMC2234137

[31] Gordon CP, Williams P, Chan WC. Attenuating *Staphylococcus aureus* virulence gene regulation: a medicinal chemistry perspective. J Med Chem. 2013;56(4):1389–404. 10.1021/jm3014635 23294220 PMC3585718

[32] Geisinger E, George EA, Muir TW, Novick RP. Identification of ligand specificity determinants in AgrC, the *Staphylococcus aureus* quorum-sensing receptor. J Biol Chem. 2008;283(14):8930–8. 10.1074/jbc.M710227200 18222919 PMC2276371

[33] Geisinger E, Muir TW, Novick RP. Agr receptor mutants reveal distinct modes of inhibition by staphylococcal autoinducing peptides. Proc Natl Acad Sci USA. 2009;106(4):1216–21. 10.1073/pnas.0807760106 19147840 PMC2633565

[34] Mayville P, Ji G, Beavis R, Yang H, Goger M, Novick RP, et al Structure-activity analysis of synthetic autoinducing thiolactone peptides from *Staphylococcus aureus* responsible for virulence. Proc Natl Acad Sci USA. 1999;96:1218–23. 9990004 10.1073/pnas.96.4.1218 PMC15443

[35] Sully EK, Malachowa N, Elmore BO, Alexander SM, Femling JK, Gray BM, et al Selective chemical inhibition of *agr* quorum sensing in *Staphylococcus aureus* promotes host defense with minimal impact on resistance. PLoS Pathogens. 2014;10(6):e1004174 10.1371/journal.ppat.1004174 24945495 PMC4055767

[36] Figueroa M, Jarmusch AK, Raja HA, El-Elimat T, Kavanaugh JS, Horswill AR, et al Polyhydroxyanthraquinones as quorum sensing inhibitors from the guttates of *Penicillium restrictum* and their analysis by Desorption Electrospray Ionization Mass Spectrometry. J Nat Prod. 2014;77(6):1351–8. 10.1021/np5000704 24911880 PMC4073659

[37] Daly SM, Elmore BO, Kavanaugh JS, Triplett KD, Figueroa M, Raja HA, et al ω-Hydroxyemodin limits *Staphylococcus aureus* quorum sensing-mediated pathogenesis and inflammation. Antimicrob Agents Chemother. 2015;59(4):2223–35. 10.1128/aac.04564-14 25645827 PMC4356798

[38] Nielsen A, Månsson M, Bojer MS, Gram L, Larsen TO, Novick RP, et al Solonamide B inhibits quorum sensing and reduces *Staphylococcus aureus* mediated killing of human neutrophils. PLoS One. 2014;9(1):e84992 10.1371/journal.pone.0084992 24416329 PMC3885660

[39] Nakayama J, Uemura Y, Nishiguchi K, Yoshimura N, Igarashi Y, Sonomoto K. Ambuic acid inhibits the biosynthesis of cyclic peptide quormones in Gram-Positive bacteria. Antimicrob Agents Chemother. 2009;53(2):580–6. 10.1128/aac.00995-08 19015326 PMC2630657

[40] WHO. World Health Organization guidelines on good agricultural and collection practices (GACP) for medicinal plants. Geneva, Switzerland: WHO; 2003.

[41] Pignatti S. Flora d'Italia. Bologna, Italy: Edizioni Edagricole; 2002.

[42] Almeida I, Costa P, Bahia MF. Evaluation of functional stability and batch-to-batch reproducibility of a *Castanea sativa* leaf extract with antioxidant activity. AAPS PharmSciTech. 2010;11(1):120–5. 10.1208/s12249-009-9360-9 20066522 PMC2850504

[43] Malik A, Yuldashev MP, Obid A, Ismoil T, Ping LY. Flavonoids of the aerial part of *Lycopus lucidus* . Chem Nat Cmpd. 2002;38(6):612–3. 10.1023/A:1022667611501

[44] M100-S23. Performance standards for antimicrobial testing; 23rd informational supplement: Clinical and Laboratory Standards Institute; 2013.

[45] M45-A2. Methods for antimicrobial dilution and disk susceptibility testing of infrequently isolated or fastidious bacteria; Approved guideline-2nd Edition: Clinical and Laboratory Standards Institute; 2010. 31339681

[46] Tsai T-H, Tsai T-H, Wu W-H, Tseng JT-P, Tsai P-J. *In vitro* antimicrobial and anti-inflammatory effects of herbs against *Propionibacterium acnes* . Food Chem. 2010;119(3):964–8. 10.1016/j.foodchem.2009.07.062

[47] Kirchdoerfer RN, Garner AL, Flack CE, Mee JM, Horswill AR, Janda KD, et al Structural basis for ligand recognition and discrimination of a quorum-quenching antibody. J Biol Chem. 2011;286(19):17351–8. 10.1074/jbc.M111.231258 21454495 PMC3089576

[48] Boles B, Thoendel M, Roth A, Horswill A. Identification of genes involved in polysaccharide-independent Staphylococcus aureus biofilm formation. PLoS One. 2010;5(e10146). 10.1371/journal.pone.0010146 PMC2854687 20418950

[49] Olson ME, Nygaard TK, Ackermann L, Watkins RL, Zurek OW, Pallister KB, et al *Staphylococcus aureus* nuclease is an SaeRS-dependent virulence factor. Infect Immun. 2013;81(4):1316–24. 10.1128/IAI.01242-12 23381999 PMC3639593

[50] Otto M, Gotz F. Analysis of quorum sensing activity in staphylococci by RP-HPLC of staphylococcal delta-toxin. Biotechniques. 2000;28(6):1088–96. 10868273 10.2144/00286bm07

[51] Somerville GA, Cockayne A, Dürr M, Peschel A, Otto M, Musser JM. Synthesis and deformylation of *Staphylococcus aureus* δ-toxin are linked to tricarboxylic acid cycle activity. J Bacteriol. 2003;185(22):6686–94. 14594843 10.1128/JB.185.22.6686-6694.2003 PMC262117

[52] Beenken KE, Blevins JS, Smeltzer MS. Mutation of *sarA* in *Staphylococcus aureus* limits biofilm formation. Infect Immun. 2003;71(7):4206–11. 12819120 10.1128/IAI.71.7.4206-4211.2003 PMC161964

[53] Gillaspy A, Hickmon S, Skinner R, Thomas J, Nelson C, Smeltzer M. Role of the accessory gene regulator (*agr*) in pathogenesis of staphylococcal osteomyelitis. Infect Immun. 1995;63(9):3373–80. 7642265 10.1128/iai.63.9.3373-3380.1995 PMC173464

[54] Talekar SJ, Chochua S, Nelson K, Klugman KP, Quave CL, Vidal JE. 220D-F2 from Rubus ulmifolius kills Streptococcus pneumoniae planktonic cells and pneumococcal biofilms. PLoS One. 2014;9(5):e97314 10.1371/journal.pone.0097314 24823499 PMC4019571

[55] Cox P, Balick M. The ethnobotanical approach to drug discovery. Sci Am. 1994;270(1):82–7. 8023119

[56] Almeida IF, Fernandes E, Lima JLFC, Costa PC, Bahia MF. Protective effect of *Castanea sativa* and *Quercus robur* leaf extracts against oxygen and nitrogen reactive species. J Photochem Photobiol B. 2008;91(2–3):87–95. 10.1016/j.jphotobiol.2008.02.001 18337113

[57] Almeida IF, Pinto AS, Monteiro C, Monteiro H, Belo L, Fernandes J, et al Protective effect of *C*. *sativa* leaf extract against UV mediated-DNA damage in a human keratinocyte cell line. J Photochem Photobiol B. 2015;144(0):28–34. 10.1016/j.jphotobiol.2015.01.010 25686820

[58] Almeida IF, Valentão P, Andrade PB, Seabra RM, Pereira TM, Amaral MH, et al *In vivo* skin irritation potential of a *Castanea sativa* (Chestnut) leaf extract, a putative natural antioxidant for topical application. Basic Clin Pharmacol. 2008;103(5):461–7. 10.1111/j.1742-7843.2008.00301.x 18793273

[59] Henry F, Danoux L, Pauly G, inventors; Cognis France S.A.S., Boussens (FR), assignee. Cosmetic compositions containing an extract of leaves of the Castanea sativa plant and cosmetic treatments USA2011.

[60] Grice E, Kong H, Conlan S, Deming C, Davis J, Young A, et al Topographical and temporal diversity of the human skin microbiome. Science. 2009;324(5931):1190–2. 10.1126/science.1171700 19478181 PMC2805064

[61] Muszer M, Noszczyńska M, Kasperkiewicz K, Skurnik M. Human microbiome: When a friend becomes an enemy. Arch Immunol Ther Exp. 2015:1–12. 10.1007/s00005-015-0332-3 PMC4499106 25682593

[62] Naik S, Bouladoux N, Linehan JL, Han S-J, Harrison OJ, Wilhelm C, et al Commensal-dendritic-cell interaction specifies a unique protective skin immune signature. Nature. 2015;520(7545):104–8. 10.1038/nature14052 25539086 PMC4667810

[63] Elfawal MA, Towler MJ, Reich NG, Weathers PJ, Rich SM. Dried whole-plant *Artemisia annua* slows evolution of malaria drug resistance and overcomes resistance to artemisinin. Proc Natl Acad Sci USA. 2015;112(3):821–6. 10.1073/pnas.1413127112 25561559 PMC4311864

[64] Schmidt BM, Ribnicky DM, Lipsky PE, Raskin I. Revisiting the ancient concept of botanical therapeutics. Nat Chem Biol. 2007;3(7):360–6. 10.1038/nchembio0707-360 17576417

[65] Novick RP, Ross HF, Projan SJ, Kornblum J, Kreiswirth B, Moghazeh S. Synthesis of staphylococcal virulence factors is controlled by a regulatory RNA molecule. Embo J. 1993;12(10):3967–75. 7691599 10.1002/j.1460-2075.1993.tb06074.x PMC413679

[66] Parker D, Narechania A, Sebra R, Deikus G, LaRussa S, Ryan C, et al Genome sequence of bacterial interference strain Staphylococcus aureus 502A. Genome Announc. 2014;2(2). 10.1128/genomeA.00284-14 PMC3983310 24723721

[67] Ibberson CB, Jones CL, Singh S, Wise MC, Hart ME, Zurawski DV, et al *Staphylococcus aureus* hyaluronidase is a CodY-regulated virulence factor. Infect Immun. 2014;82(10):4253–64. 10.1128/iai.01710-14 25069977 PMC4187871

[68] Baba T, Takeuchi F, Kuroda M, Yuzawa H, Aoki K-i, Oguchi A, et al Genome and virulence determinants of high virulence community-acquired MRSA. The Lancet. 2002;359(9320):1819–27. 10.1016/S0140-6736(02)08713-5 12044378

[69] McDougal LK, Steward CD, Killgore GE, Chaitram JM, McAllister SK, Tenover FC. Pulsed-field gel electrophoresis typing of oxacillin-resistant *Staphylococcus aureus* isolates from the United States: Establishing a national database. J Clin Microbiol. 2003;41(11):5113–20. 14605147 10.1128/JCM.41.11.5113-5120.2003 PMC262524

