# Correction: *Castanea sativa* (European Chestnut) Leaf Extracts Rich in Ursene and Oleanene Derivatives Block *Staphylococcus aureus* Virulence and Pathogenesis without Detectable Resistance

**DOI:** 10.1371/journal.pone.0163655

**Published:** 2016-09-22

**Authors:** C. L. Quave, J. T. Lyles, J. S. Kavanaugh, K. Nelson, C. P. Parlet, H. A. Crosby, K. P. Heilmann, A. R. Horswill

In the methods section “Characterization by HPLC and LC-FTMS”, the identities of solvents A and B were switched. Solvent A should be listed as 0.1% formic acid in water, not in 0.1% formic acid in acetonitrile; and vice versa for solvent B. The corrected text is indicated in bold and underlined below:

An analytical HPLC-method was developed for the purposes of characterization of 224 and fractions. The analysis was performed on an Agilent 1260 Infinity system running OpenLab CDS ChemStation (Agilent Technologies, Santa Clara, CA, USA) with an Agilent ZORBAX Eclipse XDB-C18 (250 mm x 4.6 mm, 5 μm) column with compatible guard column at a column temperature of 40°C. Mobile phase reagents were HPLC-grade and purchased from Fisher Scientific, except for the Type 1 water, which was obtained from an EMD Millipore MILLI-Q water system (Billerica, MA). Mobile phase consisted of a linear gradient elution **0.1% formic acid in water (A) and 0.1% formic acid in acetonitrile (B)** at a flow rate of 1 mL/min. Initial conditions were 98:2 (A:B) changing to 70:30 (A:B) at 50 min, to 2:98 (A:B) at 70 min and held until 85min. Samples were prepared in DMSO and 10 μL injections were made. Chromatograms were monitored at 254 nm and 314 nm.

Liquid chromatography-Fourier transform mass spectrometry (LC-FTMS) was performed on 224C-F2 using a Shimadzu SIL-ACHT and Dionex 3600SD HPLC pump with a modification of the previous chromatographic conditions. A 20 μL injection at ambient temperature with **0.1% formic acid in water (A) and 0.1% formic acid in Optima LC/MS acetonitrile (Fisher Scientific) (B)** at a flow rate of 1 mL/min. Initial conditions were 98:2 (A:B) changing to 64:36 (A:B) at 12 min, to 52:48 (A:B) at 86 min, 2:98 (A:B) at 102.6 min and held until 117.6 min before returning to initial conditions to equilibrate the column. The data was acquired in MS^1^ mode scanning from a *m/z* of 150–1500 on a Thermo Scientific LTQ-FT Ultra MS in negative ESI mode and processed with Thermo Scientific Xcalibur 2.2 SP1.48 software (San Jose, CA). The capillary temperature was 275.0°C, sheath gas of 60, source voltage and current 5.0 kV and 100.0 μA, and the capillary voltage -49.0 V.
